# Placenta-oriented self-assembled Aspirin-RGDV nanoconjugates attenuate preeclampsia through restoration of angiogenic balance

**DOI:** 10.1016/j.ijpx.2026.100545

**Published:** 2026-04-16

**Authors:** Ying Zhang, Wenqiang Qian, Yao Yao, Dongli Sun, Zhiyuan Ma, Xian Zhang, Huidi Jiang, Tian Dong, Weidong Fei, Caihong Zheng

**Affiliations:** a Research Center for Clinical Pharmacy, College of Pharmaceutical Sciences, Zhejiang University, Hangzhou 310058, China; b Women's Hospital, Zhejiang University School of Medicine, Hangzhou 310006, China

**Keywords:** Pre-eclampsia, A-RGDV, Placenta, Vessel, Nanoconjugates

## Abstract

Pre-eclampsia (PE) is a placenta-originated pregnancy disorder and a leading cause of maternal-fetal morbidity and mortality worldwide. Aspirin is the only widely recommended evidence-based prophylaxis for PE, yet its therapeutic potential is constrained by limited placental exposure and concerns with long-term dosing. To address these issues, this study engineered an aspirin-RGDV tetrapeptide conjugate (A-RGDV) that self-assembled into serum-stable nanostructures capable of selectively targeting placental integrin αV. The designed A-RGDV showed negligible hemolytic activity and no detectable cytotoxicity toward HTR-8/SVneo trophoblast cells across a concentration range of 5–200 μg/mL. Under CoCl₂ hypoxia-mimetic stress, A-RGDV improved trophoblast viability beyond equimolar aspirin and reduced total apoptosis from 34.6% (model) to 13.6%, *versus* 23.4% with aspirin. Pharmacokinetic studies revealed that nanoconjugates improved the *in vivo* behavior of aspirin, evidenced by prolonged plasma retention and an increased area under the concentration-time curve. Tissue distribution studies confirmed pronounced placental enrichment of A-RGDV, with placental drug levels increased by over 50-fold at both 2 h and 6 h compared with aspirin, while fetal exposure was markedly reduced. In an L-NAME-induced murine PE model, A-RGDV more effectively attenuated gestational hypertension than low-dose aspirin, increased fetal and placental weights, and reduced resorption rates to less than 10%. Circulating and placental angiogenic profiles trended toward rebalanced sFlt-1/PlGF, alongside correction of thromboxane/prostacyclin signaling. Collectively, A-RGDV provides integrin-guided placental protection and superior maternal-fetal benefits compared with aspirin, supporting placenta-targeted, dose-sparing therapy for PE.

## Graphical abstract

**Figure:**
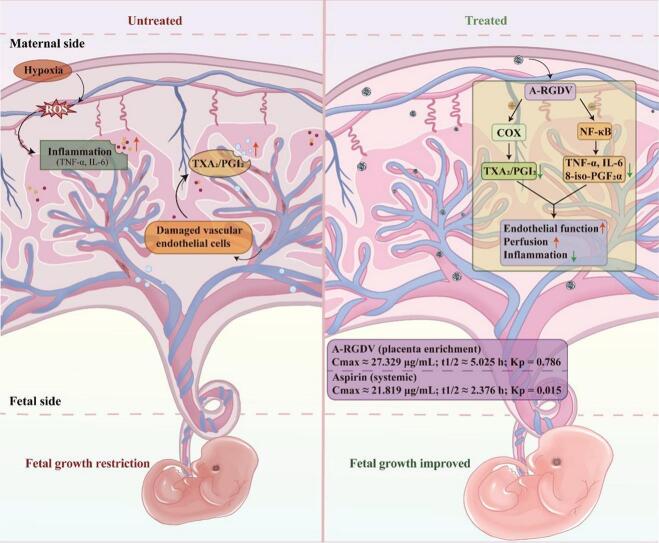

## 1 Introduction

Pre-eclampsia (PE) is a multisystem disorder characterized by new-onset hypertension after 20 weeks of gestation with proteinuria, maternal organ dysfunction, or uteroplacental compromise (Dimitriadis et al., 2023; Poon et al., 2019). It remains one of the gravest complications of pregnancy and a leading contributor to maternal and perinatal morbidity and mortality (Poon et al., 2019). Worldwide, PE is estimated to claim the lives of about 700,000 maternal and 500,000 fetal annually (Magee et al., 2022). The survivors face long-term risks of stroke, cardiovascular disease, and glucose dysmetabolism, while offspring exhibit increased rates of preterm delivery, intrauterine growth restriction, and later metabolic syndrome (Chappell et al., 2021; Pittara et al., 2021). In the context of declining fertility and increasing advanced maternal age, the public-health burden of PE is increasingly evident. Despite progress in screening and supportive care, there is still no disease-modifying drug once PE is established (Magee et al., 2022). Current management remains symptomatic antihypertensives, magnesium sulfate, and sedatives, providing limited prolongation of gestation, and delivery of the placenta remains the only definitive treatment (Magee et al., 2022; Walton et al., 2018).

The prevailing “two-stage” model posits that defective early placentation impaired decidual/spiral artery remodeling induces high shear stress, hypoxia, and oxidative stress within the intervillous space, prompting placental release of anti-angiogenic factors (*e.g.*, soluble fms-like tyrosine kinase-1, sFlt-1) and inflammatory mediators into the maternal circulation and culminating in systemic endothelial dysfunction and hypertension (Redman and Sargent, 2009; Redman et al., 2022; Staff, 2019). Thus, restoring placental angiogenesis perfusion or reducing the maternal sFlt-1 burden are rational disease-modifying strategies (Pankiewicz et al., 2021; Staff et al., 2022). Low-dose aspirin (LDA) is the only evidence-based prophylactic for high-risk women (Henderson et al., 2021; Magee et al., 2022). Mechanistically, LDA irreversibly acetylates cyclooxygenase-1 (COX-1) in platelets, suppressing thromboxane A₂ (TXA₂) while relatively sparing prostacyclin (PGI₂) to improve vascular tone and microcirculation. It also downregulates NF-κB-driven inflammatory. When initiated early in pregnancy and maintained, LDA may facilitate spiral-artery remodeling and attenuate placental inflammation (Loussert et al., 2020; Rolnik et al., 2022; Su et al., 2019). However, free aspirin shows modest placental accumulation, offers limited benefit in established disease. Meanwhile, extended aspirin administration across pregnancy is associated with maternal-fetal safety concerns, with gastrointestinal or renal adverse effects affecting adherence in ∼10% of users and an elevated risk of postpartum hemorrhage when used near term (Hastie et al., 2021; Mone et al., 2018). The transplacental exposure may attenuate neonatal platelet function and affect development (Hastie et al., 2021; Leonhardt et al., 2003). Most guidelines, therefore, recommend discontinuation by term or prior to delivery.

**Fig. 1:**
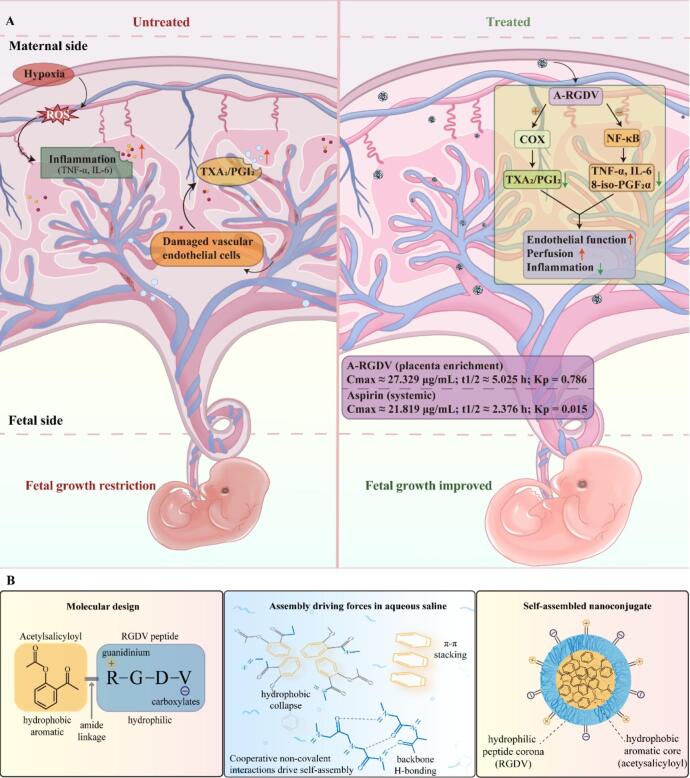
(A) Schematic diagram of treatment mechanism. (B) Self-assembly mechanism of A-RGDV nanoconjugates.

Targeted nanotherapeutic approaches offer a rational strategy to spatially confine therapeutic action to the maternal-fetal interface (Swingle et al., 2025; Zhang et al., 2023b). Our group has previously demonstrated that integrins αvβ3 and αvβ5 are highly expressed on trophoblasts and placental endothelium, and that RGD-containing peptides bind these receptors with nanomolar affinity (Tang et al., 2024; Tang et al., 2023). Based on this rationale, we developed a placenta-targeting strategy by synthesizing aspirin-RGDV peptide nanoconjugates (A-RGDV), in which the RGD motif enables integrin αv-mediated homing and cellular uptake. The conjugate self-assembles into plasma-stable nanoconjugates, thereby enhancing local drug exposure in the placenta while reducing off-target and fetal exposure. In this study, we systematically characterized the structure of A-RGDV and evaluated its cellular activity, pharmacokinetics, targeted placental distribution, therapeutic efficacy, and safety, in direct comparison with an equivalent dose of aspirin. Collectively, this work established a rational and translatable framework for placenta-oriented, dose-sparing therapy, offering a potential disease-modifying approach for the treatment of established pre-eclampsia (Fig. 1A).

## 2 Materials and methods

### 2.1 Materials

N-nitro-L-arginine methyl ester (L-NAME) was obtained from Aladdin Reagent Co., Ltd. (Shanghai, China). Sodium carboxymethyl cellulose was purchased from McLean Biochemical Technology Co., Ltd. (Shanghai, China). Protective L-configured amino acids and salicylic acid (SA) were from Sigma-Aldrich Co. LLC (St. Louis, MO, USA). All peptide coupling and deprotection reactions were performed under anhydrous conditions. Dimethyl sulfoxide (DMSO) was supplied by Sinopharm Chemical Reagent Co., Ltd. (Shanghai, China). For cell culture, RPMI-1640 (BasalMedia, Shanghai, China), trypsin-EDTA (0.25%), fetal bovine serum (FBS), phosphate-buffered saline (PBS), and penicillin-streptomycin solution (Gibco BRL, MD, USA) were used. The Cell Counting Kit-8 (CCK-8) was from Meilunbio (Dalian, China). Paraformaldehyde (4%, *v*/v) was purchased from Servicebio (Wuhan, China). 4′,6-diamidino-2-phenylindole (DAPI) was obtained from Thermo Fisher Scientific (MA, USA). ELISA kits were purchased from Shanghai Enzyme-linked Biotechnology Co., Ltd. (Shanghai, China), with the following catalog numbers: Mouse sFlt-1 (ml106745), Mouse PlGF (ml106707), Mouse VEGF (ml057559), TXB₂ (ml601808), 6-keto-PGF₁α (ml002160), 8-isoPGF₂α (ml038632), TNF-α (ml077385), and IL-6 (ml058097). All other reagents were of analytical grade.

### 2.2 Cells and animals

The normal human trophoblast cell line (HTR-8/SVneo) was acquired from the Chinese Academy of Sciences Cell Bank (Shanghai, China) and grown in RPMI-1640 with 10% FBS. Sprague-Dawley (SD) rats, Institute of Cancer Research (ICR) mice were purchased from the Beijing Vital River Laboratory Animal Technology Co., Ltd. (Beijing, China). All animal experiment protocols were approved by the Scientific Investigation Board of Zhejiang Chinese Medical University (approval number IACUC-20251110-05).

### 2.3 Preparation of aspirin-RGDV

A-RGDV was prepared on 2-chlorotrityl chloride (2-CTC) resin using standard Fmoc solid-phase peptide synthesis. After loading Fmoc-Val-OH, the tetrapeptide was assembled C → N (Asp (OtBu) → Gly → Arg (Pbf)) *via* iterative 20% piperidine deprotections and HBTU/DIEA couplings, monitoring completion by the Kaiser test. The N-terminal free amine was then acylated on-resin with aspirin (HBTU/DIEA in dry DMF) under mild, moisture-free conditions to preserve the acetyl group. Global deprotection or cleavage used TFA/EDT/TIS/H₂O (95:2:2:1), and the crude was precipitated with cold ether, dissolved, and purified by preparative reverse-phase high-performance liquid chromatography (RP-HPLC). Fractions were pooled and lyophilized to give A-RGDV (typical purity ≥85%), with identity confirmed by analytical HPLC and electrospray ionization mass spectrometry.

### 2.4 Characterization of aspirin-RGDV

Hydrodynamic diameter and polydispersity index (PDI) of A-RGDV nanoconjugates were measured by dynamic light scattering using a Zetasizer Nano ZS90 (Malvern Instruments, USA). All measurements were performed at room temperature with three independent replicates per sample. Particle morphology was assessed by transmission electron microscopy (TEM; JEM-1400, JEOL, Tokyo, Japan). Formvar-coated copper grids were immersed in the nanoparticle aqueous dispersion, air-dried at ambient temperature, and imaged at an accelerating voltage of 80–120 kV. Atomic force microscopy (AFM; Vecco, Santa Barbara, CA, USA) was conducted under ambient conditions on a Veeco Metrology system operated in contact mode to obtain topographical images. Structural identity was confirmed by nuclear magnetic resonance (NMR; Bruker, Germany) spectroscopy and mass spectrometry: conformational features were probed by two-dimensional COSY experiments, and molecular mass was verified by electrospray ionization mass spectrometry (positive mode) and time-of-flight mass spectrometry (TOF-MS; Bruker, Germany).

### 2.5 Assessment of *in vitro* stability

To evaluate the *in vitro* stability of A-RGDV nanostructures, they were dissolved in PBS (pH 7.4) or PBS solution containing 10% FBS. After incubation at 37 °C for predetermined time periods (1, 2, 3, 5, and 7 day), 1 mL suspension was taken to determine the particle size of A-RGDV. Particle size was evaluated as described above. The experiment was operated three times in parallel.

### 2.6 Hemolysis assay

Fresh anticoagulated rat blood (8 mL) was centrifuged at 3000 ×*g* for 15 min; the supernatant was discarded. Erythrocytes were washed three times with normal saline (0.9% NaCl) by centrifugation and then resuspended in saline to prepare a 2% (*v*/v) suspension. Samples were divided into five groups in triplicate: positive control (500 μL distilled water +500 μL erythrocyte suspension), negative control (500 μL saline +500 μL erythrocyte suspension), and A-RGDV groups (500 μL test formulation at 50, 100, 150, or 200 μg/mL +500 μL erythrocyte suspension). Following incubation at 37 °C for 1 h, samples were centrifuged (3000 ×*g*, 5 min), and supernatant absorbance was measured at 545 nm using a microplate reader. Hemolysis ratio = (OD**_A-RGDV_** − OD**_N_**)/ (OD**_P_** − OD**_N_**) × 100%, where OD**_A-RGDV_**, OD**_N_**, and OD**_P_** represent the optical densities of the test group, negative control, and positive control, respectively; hemolysis less than 5% indicated no significant hemolysis.

### 2.7 *In vitro* cytotoxicity

Cytotoxicity of aspirin and A-RGDV toward HTR-8/SVneo trophoblasts was assessed using the Cell Counting Kit-8 (CCK-8). Cells (4 × 10^3^ cells/well) were seeded in 96-well plates in complete growth medium and allowed to adhere for 24 h. Cultures were then exposed for 24 h to serial concentrations (5–200 μg/mL) of aspirin or A-RGDV; vehicle-matched controls and medium-only blanks were included. After treatment, 10 μL CCK-8 reagent was added to each well, and plates were incubated for 1.5 h. Absorbance was recorded at 450 nm using a microplate reader. Background (blank) values were subtracted, and cell viability was expressed as a percentage of the untreated control:

$$\text{Viability}\;\left(\%\right)=100\times \left(A₄₅₀,\text{sample}-A₄₅₀,\text{blank}\right)/\left(A₄₅₀,\text{control}-A₄₅₀,\text{blank}\right)$$

Each experiment comprised ≥3 technical replicates per concentration and was repeated independently three times.

### 2.8 Cellular uptake assay

Cellular uptake of aspirin and A-RGDV was quantified by LC-MS/MS using intracellular salicylic acid (SA) as the surrogate. HTR-8/SVneo and HUVEC cells were seeded in 6-well plates at 3 × 10^5^ cells/well and incubated overnight. Cells were then treated with aspirin or A-RGDV at an aspirin-equivalent concentration of 100 μg/mL for 0.5 h and 2 h.

After incubation, cells were washed three times with ice-cold PBS to remove extracellular drug and lysed with 200 μL of ice-cold extraction solvent (80% methanol containing 0.1% formic acid and SA-d_4_, 50 ng/mL). Cells were scraped, collected, and centrifuged at 12000 ×*g* for 10 min at 4 °C. The supernatants were collected for LC-MS/MS analysis. Matrix-matched calibration standards were prepared by spiking SA into pooled blank cell extracts processed in the same manner.

LC-MS/MS analysis was performed on a Waters ACQUITY™ Premier triple quadrupole mass spectrometer (MA, USA). Chromatographic separation was achieved on a BEH C18 column (100 × 2.1 mm, 1.7 μm) at 35 °C using isocratic elution with 0.1% (*v*/v) formic acid in water (A) and acetonitrile (B) at a ratio of 78:22. The flow rate was set at 0.3 mL/min. Quantification was carried out in multiple reaction monitoring (MRM) mode. For SA, the parent ion was *m*/*z* 137.06 and the daughter ion was *m/z* 64.94, with a cone voltage of 10 V and a collision energy of 24 eV. For SA-d_4_, the parent ion was *m/z* 141.05 and the daughter ion was *m/z* 97.03, with a cone voltage of 8 V and a collision energy of 10 eV.

### 2.9 Effects of aspirin-RGDV on migration and invasion in HTR-8/SVeno

HTR-8/SVneo trophoblasts (4 × 10^4^ cells/well) were seeded into the upper chambers of Transwell inserts. The lower chambers were filled with 300 μL RPMI-1640 medium, and the plates were incubated for 4 h at 37 °C to allow cell attachment. The upper-chamber medium was then replaced with 100 μL of medium containing either aspirin or A-RGDV for a 2 h pre-treatment, after which it was exchanged for CoCl₂-containing medium (300 μM). After 12 h, non-migrated cells on the upper surface were removed; membranes were fixed, stained with crystal violet, and migrated cells on the lower surface were quantified under a light microscope.

For Matrigel invasion, 100 μL of Matrigel diluted in serum-free medium was applied to the upper chamber and allowed to polymerize at 37 °C for 1–2 h. HTR-8/SVneo cells (4 × 10^4^ cells/well) were then seeded into the coated upper chambers; 300 μL RPMI-1640 medium was added to the lower chambers, and plates were incubated for 4 h. The upper-chamber medium was replaced with 100 μL medium containing aspirin or A-RGDV for a 2 h pre-treatment, followed by replacement with CoCl₂-containing medium (300 μM). After 12 h, residual cells and Matrigel on the upper surface were gently removed; membranes were fixed, stained with crystal violet, and invaded cells adherent to the lower surface were enumerated microscopically. For both assays in this section, at least three independent experiments were performed with technical replicates. Results were analyzed as the mean cell count per insert (averaged across ≥5 random fields) and normalized to the corresponding control.

### 2.10 *In vitro* apoptosis assay

HTR-8/SVneo trophoblasts were seeded into 6-well plates and allowed to adhere for 4 h at 37 °C (5% CO₂). Cells were then pretreated for 12 h with medium containing either aspirin or A-RGDV. Subsequently, the medium was replaced with CoCl₂-containing medium (300 μM) and incubated for a further 12 h to induce hypoxia-mimetic stress. Cells were harvested with 0.25% trypsin (without EDTA), neutralized, and collected by centrifugation (1000 ×*g*, 5 min). Pellets were washed twice with cold PBS and resuspended in binding buffer. Apoptosis was assessed using Annexin V-FITC and propidium iodide (PI) staining according to the manufacturer's instructions: cells were incubated with Annexin V-FITC and PI for 10–15 min at room temperature in the dark and analyzed immediately by flow cytometry. Experiments were performed in triplicate and repeated independently at least three times. The anti-apoptotic effects of aspirin and A-RGDV were compared to the CoCl₂-treated control.

### 2.11 *In vitro* angiogenesis assay

Matrigel (pre-chilled) was added to 96-well plates (50 μL/well) and polymerized at 37 °C for 30 min. Treated endothelial cells (2 × 10^4^ cells/well) were seeded onto the Matrigel layer in the presence of the indicated treatments (with or without CoCl₂). After 4 h, tube-like structures were imaged using an inverted microscope.

### 2.12 Determination of cell supernatant TXB_2_, 6-keto-pGF_1_α, TNF-α, IL-6, and 8-isoPGF_2_α

The cell supernatant collected from different treatment groups was centrifuged (1000 ×*g*, 5 min), and the supernatant was collected and stored at −80 °C. The TXB_2_, 6-keto-pGF_1_α, TNF-α, IL-6, and 8-isoPGF_2_α levels of cell supernatant were determined by ELISA kits. All operations were performed according to the instructions of the kits.

### 2.13 Pharmacokinetic study

Female SD rats (220–250 g) were acclimated for one week at 25 ± 2 °C before the experiment. Animals were randomly assigned to three groups, each consisting of five rats. Aspirin or A-RGDV was administered either by oral gavage or intravenous injection at a dose of 10 mg/kg. Blood samples were collected from the central retinal vein at 0.25, 0.75, 1, 1.5, 2, 4, 8, 10, 12, and 24 h after dosing. Plasma was separated by centrifugation at 4000 ×*g* for 15 min and stored for subsequent analysis. Plasma proteins were precipitated with methanol, and drug concentrations were determined by liquid chromatography-mass spectrometry (LC-MS, Bruker, Germany). Pharmacokinetic parameters, including half-life (t_1/2_), maximum plasma concentration (C_max_), clearance (CL/F), apparent volume of distribution (Vz/F), and area under the concentration–time curve (AUC), were calculated using Phoenix software.

Chromatographic separation was performed on a Phenomenex ACE Excel C18-AR column (250 × 4.6 mm, 5 μm). The mobile phase consisted of methanol (A) and 0.1% formic acid in water (B) at a ratio of 85:15 (*v*/v). The flow rate was 0.3 mL/min, the column temperature was 40 °C, the injection volume was 10 μL, and the total run time was 5 min.

Mass spectrometric detection employed an electrospray ionization source and ultra-fast multiple reaction monitoring (MRM) for compound identification and quantification. The nebulizing gas flow was 3.0 L/min, with drying gas and heating gas each at 10.0 L/min. The interface voltage was 4.0 kV. The interface temperature, desolvation (solvent) temperature, and heating block temperature were 300 °C, 250 °C, and 400 °C, respectively. The detector voltage was 2.22 kV. The MRM transitions used for quantification were *m/z* 137.0 → 93.0 for salicylic acid and *m/z* 141.0 → 97.0 for SA-d_4_ (internal standard).

### 2.14 Establishment of the pre-eclampsia mice

Sexually mature ICR mice (∼8 weeks old) were paired for mating at a female-to-male ratio of 2:1. The presence of a vaginal plug the following morning was recorded as 0.5 days post coitum (d.p.c.); subsequent gestational days were designated 1.5 d.p.c., 2.5 d.p.c., *etc.* Animals were maintained in the institutional animal facility under standard conditions (25 ± 2 °C; 50 ± 10% relative humidity; 12-h light/dark cycle) with *ad libitum* access to food and water.

Pre-eclampsia mice were induced by daily intraperitoneal administration of L-NAME (75 mg/kg/day) from 6.5 to 17.5 d.p.c. Confirmed pregnant mice were randomly assigned to four groups: (i) control, (ii) L-NAME (saline only), (iii) L-NAME + aspirin (oral gavage), and (iv) L-NAME + A-RGDV (intravenous, tail vein). Pharmacologic treatments (aspirin or A-RGDV) were administered once daily from 8.5 to 17.5 d.p.c., concurrent with continued L-NAME dosing in the relevant groups. At 18.5 d.p.c., pregnant mice were euthanized, maternal blood was collected, and maternal organs, placentae, and embryos were harvested and weighed. Tissues were fixed in 4% paraformaldehyde and processed for paraffin embedding and sectioning for histological analyses. All procedures were conducted in accordance with institutional guidelines for animal care and use.

### 2.15 Pharmacodynamics study

At 6.5 d.p.c., pregnant ICR mice were randomly allocated to four groups (n = 10/group): (i) normal pregnant control (no treatment), and three groups receiving daily intraperitoneal L-NAME (75 mg·kg^−1^·day^−1^) from 6.5 d.p.c. to induce a preeclampsia-like phenotype. From 8.5 d.p.c., the L-NAME-treated groups received: (ii) saline, (iii) low-dose aspirin (10 mg·kg^−1^·day^−1^; oral gavage), or (iv) A-RGDV (*i.v.*). The body weight of ICR pregnant mice was recorded every other day beginning at 6.5 d.p.c. The blood pressure was measured at 6.5, 9.5, 11.5, 13.5, 15.5, and 17.5 d.p.c. using a non-invasive tail-cuff system (photoelectric volume-pulse plethysmography). Mice were warmed and acclimated prior to acquisition; ≥ 5 consecutive readings per animal were obtained and averaged for analysis. At 18.5 d.p.c., maternal blood was collected, allowed to clot at room temperature for 1 h, and centrifuged at 3000 ×*g* for 15 min to obtain serum, which was stored at −80 °C for subsequent assays. After euthanasia, fetuses, placentae, and maternal organs were harvested. Total and viable embryos per pregnant mouse were recorded. Fetuses and placentae were rinsed in PBS, gently blotted dry, and weighed individually.

### 2.16 Determination of serum sFlt-1, PIGF, VEGF, TXB_2_, and 6-keto-pGF_1_α

The blood samples collected from pregnant mice at 18.5 d.p.c. were centrifuged (2400 ×*g*, 10 min), and the serum was collected and stored at −80 °C. The sFlt-1, PlGF, VEGF, TXB_2_, and 6-keto-pGF_1_α levels of plasma were determined by ELISA kits. All operations were performed according to the instructions of the kits.

### 2.17 Determination of in the placenta sFlt-1, PIGF of pregnant mice

The placentas and embryos collected from pregnant mice at 18.5 d.p.c. were rinsed with precooled PBS. Four or more placentas of different groups were used in this study. The tissue was cut into pieces, and 100 mg of the tissue fragment was added to a grinding tube. Two steel balls and 1 mL PBS were added to the grinding tube. The tube was then ground at 60 Hz for 10 min in a high-speed tissue grinder. The homogenate obtained after grinding was centrifuged at 21580 ×*g* for 10 min. Then, the sFlt-1 and PIGF concentration in the supernatant was determined using an ELISA kit. All operations were performed according to the instructions of the kits.

### 2.18 H&E-stained histology images of placental tissue

Placentae collected at 18.5 days of gestation were fixed in 4% paraformaldehyde, sectioned at 2 μm, and stained with hematoxylin and eosin (H&E). By observing pathological features such as inflammatory response, cellular degeneration and tissue damage, the restorative effect of drug therapy on the placenta was comprehensively assessed.

### 2.19 Immunofluorescence staining of placental tissue

Placentae were collected at 18.5 d.p.c., fixed in 4% paraformaldehyde (4 °C, overnight), dehydrated, paraffin-embedded, and sectioned at 4–5 μm. Sections were deparaffinized, rehydrated, and subjected to heat-induced antigen retrieval (10 mM citrate buffer, pH 6.0, 95–98 °C, 15 min). After cooling, slides were permeabilized with 0.2% Triton X-100 in PBS (10 min) and blocked with 5% normal serum (species matched to secondary antibody) in PBS (30 min, RT). Apoptotic nuclei were detected using a terminal deoxynucleotidyl transferase (TdT)-mediated dUTP nick-end labeling kit according to the manufacturer's protocol. For immunostaining, sections were incubated with primary antibodies against CD31 (endothelium) and α-SMA (vascular smooth muscle/pericytes) diluted in blocking buffer (4 °C, overnight), followed by species-appropriate fluorophore-conjugated secondary antibodies (1 h, RT, protected from light).

For TUNEL, apoptotic index was calculated as TUNEL^+^/DAPI^+^ nuclei (%) in ≥5 random high-power fields per section. Vascularization was assessed as CD31^+^ area fraction, and vascular maturation as α-SMA coverage (%) over CD31^+^ vessels, using ImageJ with consistent thresholds. At least three placentas per group and ≥ 2 sections per placenta were analyzed by a blinded assessor.

### 2.20 Safety evaluation of aspirin-RGDV

Safety of the formulations was evaluated using serum samples from each group of pregnant mice. An automated clinical chemistry analyzer was used to determine plasma levels of alanine aminotransferase (ALT), aspartate aminotransferase (AST), creatinine (Cr), and blood urea nitrogen (BUN). Plasma concentrations of tumor necrosis factor-α (TNF-α) and interleukin-6 (IL-6) were quantified using ELISA kits. All procedures were performed in strict accordance with the manufacturer's instructions. Representative organs (heart, liver, spleen, lung, and kidney) were fixed in 4% paraformaldehyde then subjected to H&E staining for microscopic pathology evaluation. Pathological features such as inflammation, cellular degeneration, and tissue damage were examined to comprehensively assess the *in vivo* safety profile of the drug treatment.

### 2.21 Statistical analysis

Data were presented as mean ± SD and plotted using GraphPad Prism 8.0. Group differences were analyzed by one-way ANOVA with SPSS software (Version 25.0; IBM Corp., Armonk, NY, USA). The Kruskal-Wallis H test was used instead when there was a non-normal distribution or uneven variance. Flow cytometry data were analyzed with CytExpert 2.6 software and FlowJo 8.0. *P*-values (*p*) < 0.05 were considered statistically significant, and extreme significance was set at *p* < 0.01.

## 3 Results and discussions

### 3.1 Preparation and characterization of A-RGDV

**Fig. 2:**
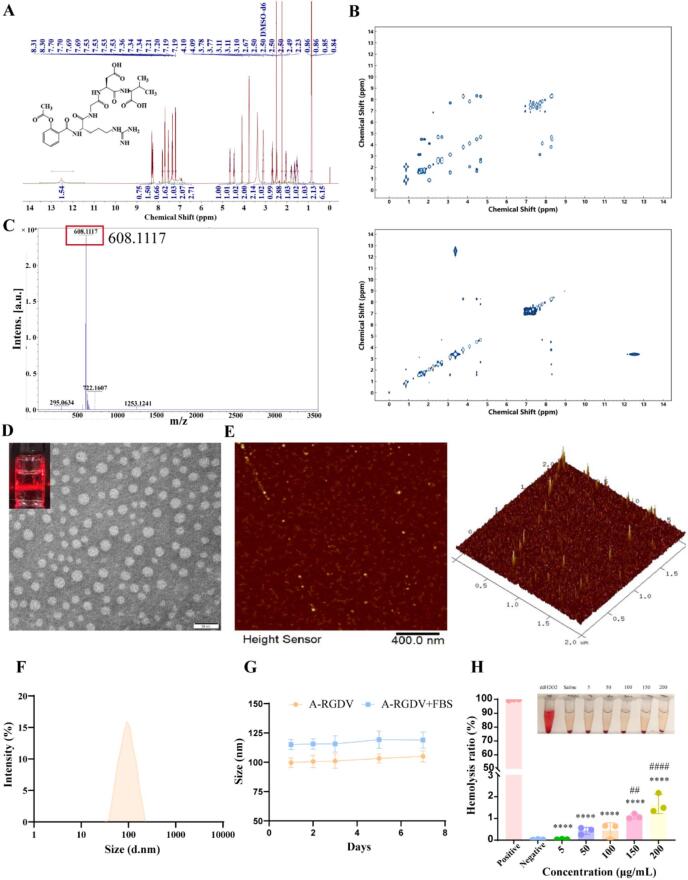
(A) ^1^H NMR spectra of A-RGDV. (B) COSY NMR spectra of A-RGDV. (C) TOF-MS spectra of A-RGDV. (D) TEM image of A-RGDV. (E) AFM image of A-RGDV. (F) The particle size distribution of A-RGDV. (G) The particle size change curve of A-RGDV in PBS containing 10% FBS for 7 days (n = 3). (H) The hemolysis ratio of A-RGDV (n = 3). Data are presented as mean ± SD, **** *p* < 0.0001 *vs* Positive group, ## *p* < 0.01, #### *p* < 0.0001 *vs* Negative group.

A-RGDV is an amphiphilic peptide–drug conjugate formed by covalent linkage of an acetylsalicyloyl (aspirin) moiety to the RGDV tetrapeptide. Upon dispersion in aqueous media, the conjugate self-assembles into nanoscale structures driven by hydrophobic collapse and π-π interactions of the aspirin segment, together with backbone hydrogen bonding and electrostatic stabilization contributed by peptide residues, resulting in carrier-free nanoconjugates that present the RGDV motif on the surface for integrin-mediated placental targeting (Fig. 1B). The A-RGDV was successfully synthesized and unambiguously identified by multinuclear NMR and high-resolution MS. The ^1^H NMR spectrum exhibited the diagnostic features of a salicyl-acylated peptide: the acetyl signal of the acetylsalicyloyl fragment appeared as a characteristic singlet at δ∼2.23 ppm, supporting retention of the acetyl group after N-terminal acylation. In the upfield region, the Val isopropyl methyl protons were observed at δ∼0.84–0.86 ppm, consistent with the presence of the Val residue. Multiple resonances in the δ∼3.7–4.7 ppm window (∼4.65/4.46/4.10 ppm) were assigned to the Gly CH_2_ and backbone α-CH protons of the peptide residues (Val/Asp/Arg), which typically cluster in this region. The broad aliphatic region spanning δ∼1.4–3.3 ppm contained multiplets attributable mainly to Arg side-chain methylenes together with overlapping peptide aliphatic signals. In the downfield region, a cluster of resonances distributed across δ∼7.19–8.31 ppm corresponded to the aromatic protons of the aspirin ring, consistent with incorporation of the acetylsalicyloyl moiety. The residual solvent peak of DMSO‑*d*_6_ at δ∼2.50 ppm was explicitly identified and excluded from structural assignment, avoiding ambiguity in the aliphatic region (Fig. 2A). Beyond 1D chemical shifts, the 2D COSY spectra provided complementary evidence by revealing characteristic scalar-coupling networks for both the peptide segment and the aspirin aromatic ring (Fig. 2B). In the aliphatic/backbone region (∼0.8–5 ppm), COSY displayed cross-peak clusters consistent with residue-specific spin systems, including correlations expected for Val methyl ↔ Val methine, as well as coupling patterns consistent with Asp α-H ↔ β-CH and Arg α-H ↔ side-chain methylenes, supporting the presence and connectivity of the RGDV peptide framework. In the aromatic region (∼7–8.4 ppm), COSY showed a dense cluster of cross-peaks reflecting the aromatic proton coupling network, consistent with the aspirin-derived benzene ring. Collectively, the agreement between the diagnostic ^1^H resonances (acetyl, aromatic, and residue-specific aliphatic signals) and the COSY coupling patterns provides a more complete and evidence-based structural verification of A-RGDV, supporting successful N-terminal acylation by the acetylsalicyloyl group and correct assembly of the RGDV peptide segment. Electrospray TOF-MS showed a dominant ion at *m*/*z* 608.17, consistent with [M + H] ^+^ for A-RGDV; minor signals correspond to expected adducts/aggregates (*e.g.*, ∼*m/z* 626.96 [M + Na] ^+^ and ∼ *m/z* 1215 [2 M + H] ^+^) (Fig. 2C, Fig. S3). Analytical RP-HPLC (C18, 0.1% TFA in H₂O/ACN, 220 nm) displayed a sharp, baseline-resolved principal peak at t_r ≈ 9.43 min, accounting for 88.74% of total area, with two small neighbors at 8.73 min (10.24%) and 9.68 min (1.03%), indicating high chemical homogeneity and efficient purification (Fig. S2).

In saline medium, the hydrophobic acetylsalicylic acid head and the polar RGDV peptide tail spontaneously bind in water. With the help of π-π stacking of aromatic rings, backbone hydrogen bonding, and Arg-carboxylic acid electrostatic pairing, the salicylic acid moiety hydrophobically collapsed into a core, which facilitated the self-assembly of A-RGDV nanostructures. TEM demonstrated uniformly dispersed, near-spherical particles in the few-tens-of-nanometers range without obvious aggregation (Fig. 2D; inset shows Tyndall scattering), while AFM height maps (2D/3D) showed sparsely distributed nano-domains a few nanometers in height on mica (Fig. 2E), consistent with adsorbed peptide nanostructures and corroborating the lateral dimensions seen by TEM. Meanwhile, the average particle size of A-RGDV could be obtained to be close to 100 ± 3 nm based on Malvern particle sizer measurements (Fig. 2F). It should be noted that the larger particle sizes measured by DLS reflect hydrated hydrodynamic diameters in solution, in contrast to the dry-state sizes obtained by TEM and AFM (Filippov et al., 2023). Given that DLS intensity scales with diameter to the sixth power, a small fraction of larger species can disproportionately shift the intensity-weighted mean. The near constant size over 7 days in 10% FBS supported a stable hydrated architecture under physiologically relevant conditions rather than colloidal instability (Fig. 2G). Collectively, orthogonal spectroscopic, chromatographic, and microscopic data confirmed the successful synthesis of A-RGDV, its high purity and structural integrity, and the formation of stable, monodisperse peptide-based nanoconjugates.

A-RGDV exhibited excellent blood compatibility across the tested concentration range. Relative to the positive control (complete hemolysis with ddH_2_O), supernatants from all A-RGDV samples remained almost colorless, and calculated hemolysis percentages were minimal and comparable to the negative control, remaining well below the commonly accepted safety threshold of 5% for *i.v.* candidates (Fig. 2H). Given that the animal dose used in this study was derived from the clinically applied low-dose aspirin regimen for PE prevention, these findings further support the favorable hemocompatibility of A-RGDV within a translationally relevant exposure range. These data indicated that A-RGDV was unlikely to cause erythrocyte damage at pharmacologically relevant doses.

### 3.2 Cytotoxicity

**Fig. 3:**
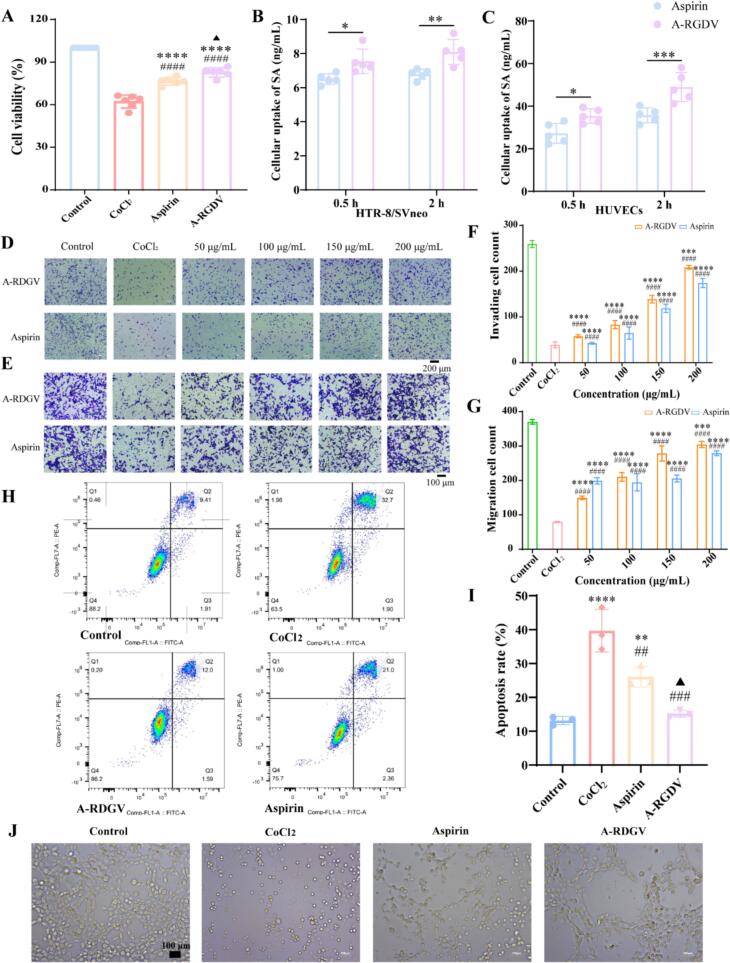
(A) Cell viability of HTR-8/SVneo cells following CoCl₂ exposure under different treatments (n = 6). Cellular uptake of aspirin and A-RGDV in (B) HTR-8/SVneo cells and (C) HUVECs (n = 5). Representative micrographs of HTR-8/SVneo cells horizontal (D) invasion and (E) migration after CoCl₂ exposure. Quantitative analysis of HTR-8/SVneo cells (F) invasion and (G) migration. (H) Representative flow-cytometry plots of Annexin V–FITC/PI staining. (I) Quantification of total apoptotic cells (n = 3). (J) Angiogenesis assay. Data are presented as mean ± SD, ** *p* < 0.01, **** *p* < 0.0001 *vs* Control group, ## *p* < 0.01, ### *p* < 0.001 *vs* CoCl_2_ group, ▲ *p* < 0.05 *vs* Aspirin-treatment group.

CCK-8 assays demonstrated that neither aspirin nor A-RGDV nanostructures was cytotoxic to HTR-8/SVneo cells over 5–200 μg /mL, mean viabilities at all doses remained ≥90% of the untreated control with no evident dose-dependent decline (Fig. S4). As shown in Fig. 3A, the CoCl₂-induced hypoxic stress markedly reduced HTR-8/SVneo cell viability compared with the control group. Treatment with free aspirin partially rescued cell viability. Under hypoxia, A-RGDV more effectively preserved viability, reduced apoptosis, and restored angiogenic behaviors than aspirin (*p* < 0.05). These data indicated that A-RGDV conferred enhanced cytoprotective effects against hypoxia-induced trophoblast injury.

### 3.3 Cellular uptake

As shown in Fig. 3B-C, A-RGDV exhibited significantly higher cellular uptake than free aspirin in both HTR-8/SVneo trophoblasts and HUVECs at 0.5 h and 2 h (*p* < 0.5). In HTR-8/SVneo cells, A-RGDV produced a modest but consistent increase in intracellular SA relative to aspirin at both time points, indicating that the uptake advantage was established early and maintained over time. In HUVECs, this difference was more pronounced, with A-RGDV showing substantially greater intracellular accumulation than aspirin at both 0.5 h and 2 h, and the gap further increased at 2 h (*p* < 0.001). The absolute uptake levels observed in HUVECs were markedly higher than those in HTR-8/SVneo cells, suggesting that endothelial cells may be particularly responsive to the RGDV-guided delivery design. Nevertheless, because trophoblast dysfunction is central to the pathogenesis of PE, HTR-8/SVneo cells were retained as the principal *in vitro* model for subsequent mechanistic evaluation. These data indicate that A-RGDV enhances aspirin-related intracellular exposure in both trophoblasts and endothelial cells. This finding is mechanistically relevant to PE, as endothelial dysfunction and impaired placental vascular remodeling are key features of disease progression, and may partly explain the superior effects of A-RGDV on angiogenic balance, vascular function, and placental protection relative to free aspirin.

### 3.4 Cell migration and cell invasion

Matrigel invasion assays showed dose-dependent enhancement of invasion by both treatments, with A-RGDV consistently outperforming aspirin across the dose range. At higher concentrations, A-RGDV restored or exceeded invaded-cell counts relative to the corresponding control, while aspirin exhibited a flatter response with larger acellular gaps (Fig. 3D and Fig. 3F). Transwell migration assays revealed a dose-responsive increase in motility for both agents. However, at every matched concentration, A-RGDV produced a visibly higher density of crystal-violet stained cells on the underside of the membrane. Notably, low-dose A-RGDV already exceeded aspirin (*p* < 0.0001), indicating greater potency, and at intermediate or high doses, the migrated area with A-RGDV approached control group, whereas aspirin produced a more modest effect (Fig. 3E and Fig. 3G).

Trophoblast migration and invasion are pivotal to early placentation. Anchoring-column cells expand and differentiate into endovascular extravillous trophoblasts (EVTs) that enter spiral arteries (Kam et al., 1999). When this process is insufficient, a cascade of low/intermittent intervillous perfusion, hypoxia-reoxygenation, and oxidative/inflammatory stress ensues, culminating in a preeclampsia-like phenotype (Burton and Hung, 2003). In this study, A-RGDV demonstrated stronger pro-migration and pro-invasion effects than equimolar aspirin *in vitro*, supporting a role in enhancing EVT function and spiral-artery remodeling, thereby improving placental perfusion and mitigating PE-related pathology.

Together with the negligible hemolysis observed for A-RGDV, these data indicated good hemocompatibility, superior pro-migratory, and pro-invasive activity of A-RGDV compared with aspirin alone. The augmented functionality is plausibly attributable to integrin engagement by the RGDV motif, which may facilitate trophoblast motility and matrix penetration under stress conditions. Collectively, the absence of toxicity, favorable hemocompatibility, and robust, dose-responsive bioactivity consistent with the compound's verified identity, purity, and colloidal stability support advancing A-RGDV for mechanistic studies and *in vivo* efficacy evaluation as a placenta-targeted therapeutic candidate.

### 3.5 Apoptosis assay

Excessive trophoblast apoptosis and insufficient trophoblast invasion are recognized as central features of placental dysfunction in PE, because they impair spiral artery remodeling and contribute to placental hypoperfusion, hypoxia, and downstream maternal endothelial injury (Torres-Torres et al., 2024; Zhang et al., 2023a). As shown in Fig. 3H-I, compared with the control group (total apoptosis, 11.3%), CoCl₂ treatment increased apoptosis to 34.6%, confirming successful induction of hypoxia-mimetic stress. Both treatments attenuated cell death, with aspirin reducing apoptosis to 23.4% and A-RGDV producing a greater reduction to 13.6%. Notably, A-RGDV conferred greater protection than aspirin (*p* < 0.05), yielding 2-fold fewer apoptotic cells *versus* the model group and markedly improving viability. This finding is particularly meaningful because trophoblast survival and invasive competence are tightly coupled in the establishment of normal placental vascular adaptation. Previous studies have shown that aspirin can promote trophoblast function, including enhanced invasion and reduced sFlt-1 production, supporting the concept that part of its benefit in PE may derive from direct placental cellular effects rather than platelet inhibition alone (Chen et al., 2025; Su et al., 2019). In our study, A-RGDV consistently outperformed aspirin at the same dose, suggesting that conjugation with the RGDV motif enhances the functional impact of aspirin under trophoblast stress conditions. Given the established role of integrin αV related signaling in trophoblast adhesion, migration, and interaction with the extracellular matrix (Johnson et al., 2023), the superior anti-apoptotic activity of A-RGDV is plausibly explained by a dual mechanism: retention of aspirin's anti-inflammatory pharmacology together with improved trophoblast engagement mediated by the RGDV sequence.

The reduction in hypoxia-induced trophoblast apoptosis was accompanied by improved migration and invasion, indicating a coordinated restoration of trophoblast function rather than a purely cytoprotective effect. This integrated phenotype is highly relevant to PE pathogenesis, because successful placentation depends not only on trophoblast survival but also on the ability of EVTs to invade and remodel maternal spiral arteries (Torres-Torres et al., 2024). Therefore, the superior anti-apoptotic effect of A-RGDV provides mechanistic support for its downstream *in vivo* benefits, linking placenta-oriented delivery to improved trophoblast fitness, enhanced vascular adaptation, and ultimately better therapeutic performance than free aspirin alone.

### 3.6 Angiogenesis assay

The *in vitro* endothelial tube formation assay recapitulated key aspects of placental and microvascular angiogenesis *in vivo*, particularly the ability of endothelial cells to undergo coordinated migration, alignment, and branching to form capillary-like networks under hypoxic stress conditions. Using CoCl₂ to chemically mimic hypoxia, we observed a marked disruption of endothelial morphogenesis, characterized by sparse, fragmented structures and loss of interconnected networks, indicating severe impairment of angiogenic capacity. Treatment with aspirin partially restored tube formation, as evidenced by increased cellular alignment and re-emergence of short tube-like segments; however, the resulting networks remained discontinuous and poorly branched. In contrast, A-RGDV treatment robustly rescued angiogenic behavior, producing a more continuous and interconnected capillary-like network with clearly defined branching points and improved structural integrity. Overall, the A-RGDV group more closely resembled the normoxic control than the aspirin group (Fig. 3J).

From a mechanistic perspective, these findings suggest that while aspirin alleviates hypoxia-associated endothelial dysfunction, targeted delivery *via* the RGDV motif confers additional benefits by enhancing endothelial engagement and local bioactivity at integrin-rich angiogenic sites. The superior network continuity and branching observed with A-RGDV are consistent with improved endothelial survival, preserved cytoskeletal organization, and restoration of angiogenesis-related cellular behaviors under hypoxia-like conditions. Collectively, these results demonstrated that A-RGDV provided enhanced protection against CoCl₂-induced angiogenic impairment, supporting its therapeutic potential for restoring vascular function in hypoxia-associated placental injury.

### 3.7 Coordinated remodeling of the prostanoid pathway and the inflammatory axis

**Fig. 4:**
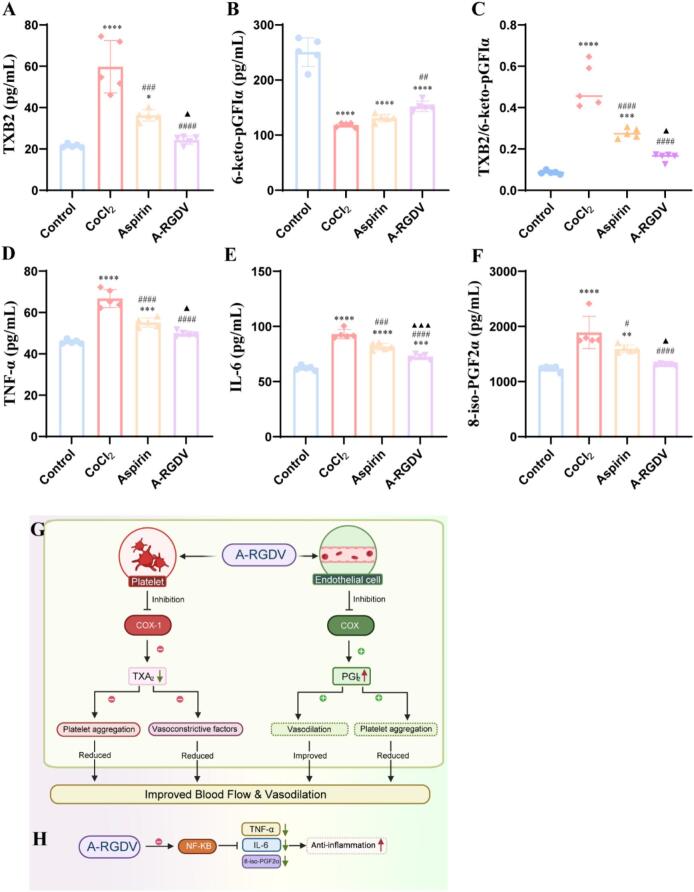
The concentration of (A) TXB_2_, (B) 6-keto-pGFIα, and (C) TXB_2_/ 6-keto-pGFIα in cell supernatant in different groups (n = 5). The concentration of (D) TNF-α, (E) IL-6, and (F) 8-isoPGF2α in serum in different groups (n = 5). (G) Vasodilatory Mechanism of A-RGDV. (H) Anti-inflammatory mechanism of A-RGDV. Data are presented as mean ± SD, * *p* < 0.05, ** *p* < 0.01, *** *p* < 0.001, **** *p* < 0.0001 *vs* Control group, # *p* < 0.05, ## *p* < 0.01, ### *p* < 0.001, #### *p* < 0.0001 *vs* L-NAME group, ▲ *p* < 0.05, ▲▲▲ *p* < 0.001 *vs* Aspirin-treatment group.

In PE, a shift toward thromboxane-dominant vasoconstrictive signaling together with reduced prostacyclin bioactivity is considered a major contributor to endothelial dysfunction, increased vascular resistance, and impaired uteroplacental perfusion. This prostanoid imbalance is closely linked to oxidative stress and lipid peroxidation, which further amplify placental and vascular injury (Walsh, 2004). In the HTR-8 hypoxia model, compared with the control group, TXB₂ increased while 6-keto-PGF₁α decreased, resulting in a marked rise in the TXB₂/6-keto-PGF₁α ratio (Fig. 4A-C). TNF-α and IL-6 were also elevated, and 8-isoPGF₂α increased, indicating a prostanoid shift toward vasoconstriction together with activation of NF-κB-related inflammation and enhanced lipid peroxidation. After aspirin treatment, TXB₂ declined, and 6-keto-PGF₁α partially recovered, thereby lowering the ratio. Inflammatory cytokines and 8-isoPGF₂α decreased in parallel, suggesting that inhibition of platelet COX-1 reduced TXA₂ production while relatively preserving endothelial PGI₂ and attenuating NF-κB-mediated inflammation and oxidative stress. A-RGDV produced stronger effects across these endpoints: lower TXB₂, higher 6-keto-PGF₁α, the lowest ratio, and more pronounced reductions in TNF-α, IL-6, and 8-isoPGF₂α (Fig. 4D-F). These data suggested that A-RGDV decreases TXA₂ *via* platelet COX-1 inhibition while preserving or enhancing PGI₂-mediated IP-cAMP-PKA signaling, thereby facilitating vascular smooth-muscle relaxation (Fig. 4G). Concurrent suppression of NF-κB signaling further contributes to reduced inflammation and oxidative stress (Fig. 4H). These findings suggest that A-RGDV not only retains the established prostanoid-regulating activity of aspirin but also enhances its functional impact under placental stress conditions (Mirabito Colafella et al., 2020). This enhanced efficacy is likely related to the placenta-oriented delivery profile of A-RGDV. Rather than acting solely as a freely distributed small molecule, A-RGDV is designed to increase local aspirin-related bioactivity at the maternal–fetal interface through RGDV-mediated placental engagement and sustained exposure. Within this framework, stronger restoration of prostanoid balance is especially meaningful, because reduced thromboxane bias together with relative preservation of prostacyclin signaling would be expected to alleviate vasoconstriction and improve local perfusion. At the same time, suppression of inflammatory cytokines and 8-isoPGF₂α suggests that A-RGDV interrupts the feed-forward cycle linking placental stress, oxidative injury, and endothelial dysfunction. These integrated biochemical effects provide a mechanistic explanation for why A-RGDV outperformed equimolar aspirin in our model.

### 3.8 Pharmacokinetic assay

**Fig. 5:**
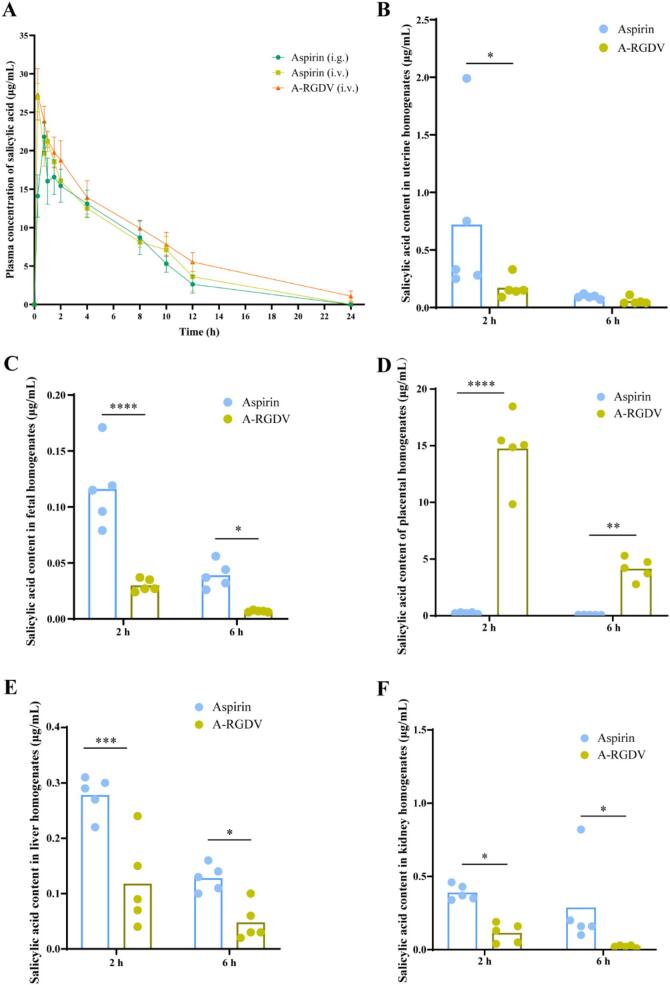
(A) Plasma drug concentration-time curve of rats (n = 5). Concentration of salicylic acid in the (B) uterus, (C) fetus, (D) placenta, (E) liver, and (F) kidney 2 h and 6 h after administration of the drug (n = 5). Data are presented as mean ± SD, * *p* < 0.05, ** *p* < 0.01, *** *p* < 0.001, **** *p* < 0.0001.

Aspirin (acetylsalicylic acid, ASA) is difficult to quantify in blood because it undergoes rapid hydrolysis after administration. A PK/PD study in pregnant women explicitly stated that salicylic acid (SA) served as a surrogate for aspirin exposure, and this approach was used to overcome the difficulty of detecting aspirin in serum due to its rapid hydrolysis within 1 h of ingestion (Vinogradov et al., 2025). For the reasons outlined above, SA was quantified by LC-MS/MS as a surrogate of aspirin exposure, and NCA was performed using SA concentration-time data. Fig. 5A showed plasma concentration-time profiles over 24 h in SD rats. Plasma salicylic acid decreased over time in all groups, while the peak characteristics and elimination phase differed due to the dosing route and formulation. Only the oral aspirin group reached the peak at a later time point, for the *i.v.* groups, the peak appeared at the earliest sampling time because the drug entered the circulation immediately after injection, followed by the elimination phase. Based on the non-compartmental analysis (NCA) parameters in Table 1, the peak concentrations in the A-RGDV *i.v.* group were close to those in the aspirin *i.v.* group (C_max_: 27.329 ± 1.319 *vs* 26.904 ± 0.726 μg/mL), suggesting that the advantage of A-RGDV does not arise from an early “higher peak”, but rather is primarily in the maintenance of the terminal phase. Compared with the aspirin *i.v.* group, A-RGDV showed a 31.2% increase in AUC_0-t_ to 185.081 ± 5.108 μg·h/mL, and a 3.24-fold prolongation of t_1/2_ to 5.025 ± 0.607 h, compared with 1.551 ± 0.069 h. This was accompanied by a significant decrease in CL/F and an increase in Vz/F (*p* < 0.05). Because salicylic acid is a metabolite, these apparent changes likely reflect combined effects of formation (release/hydrolysis), distribution, and elimination. Therefore, a more appropriate interpretation is that A-RGDV reshapes the time course of aspirin-related exposure, producing a more sustained profile rather than simply increasing the peak. Such a “similar peak but longer tail” profile may be advantageous for placenta-targeted strategies by extending the effective exposure window while potentially avoiding excessive systemic peak levels.

**Table 1:** Analysis of pharmacokinetic parameters (mean ± SD, n = 5).

Parameters	Aspirin (*i.g*.)	Aspirin (*i.v.*)	A-RGDV (*i.v.*)
t_1/2_ (h)	2.376 ± 0.419	1.551 ± 0.069 *	5.025 ± 0.607 ** ##
C_max_ (μg/mL)	21.819 ± 0.788	26.904 ± 0.726 *	27.329 ± 1.319 *
AUC_0-t_ (μ·h/mL)	124.167 ± 3.793	141.096 ± 3.087 *	185.081 ± 5.108 * #
Vz/F (L/kg)	0.256 ± 0.042	0.159 ± 0.008	0.374 ± 0.036 #
CL/F (L/h/kg)	0.075 ± 0.003	0.071 ± 0.002	0.052 ± 0.002 #

* *p* < 0.05, ** *p* < 0.01 *vs* Aspirin (*i.g.*), # *p* < 0.05, ## *p* < 0.01 *vs* Aspirin (*i.v.*)

From a therapeutic perspective, such exposure reshaping is particularly relevant to preeclampsia, where sustained modulation of platelet COX-1 activity, prostanoid balance, and vascular inflammation is required over time rather than short-lived peak effects. By extending the effective exposure window while avoiding excessive peak concentrations, A-RGDV may enhance vascular and placental protection, improve TXA₂/PGI₂ homeostasis, and reduce the risk of dose-related systemic adverse effects. These pharmacokinetic advantages provide a mechanistic basis for the superior efficacy of A-RGDV observed in downstream pharmacodynamic and therapeutic outcomes in PE models.

### 3.9 *In vivo* biodistribution assay

Building on the more sustained plasma exposure profile, this study further examined salicylic acid distribution at the maternal-fetal interface and in major organs (Fig. 5B-F). A-RGDV exhibited pronounced placental enrichment, with placental salicylic acid concentrations reaching 14.740 ± 3.105 μg/mL at 2 h, compared with 0.248 ± 0.064 μg/mL for aspirin (59.44-fold increase), and remaining at 4.146 ± 0.964 μg/mL at 6 h *versus* 0.078 ± 0.011 μg/mL (53.15-fold increase). The large and persistent increase in placental exposure suggests that A-RGDV does not merely elevate systemic exposure but likely drives a redistribution toward the placenta.

More importantly, A-RGDV did not cause a concomitant increase in fetal or major non-target organ exposure. Compared with aspirin, fetal salicylic acid levels were reduced by approximately 74.1% and 82.6% at 2 h and 6 h, respectively. In parallel, salicylic acid exposure was also lower in non-target organs, decreasing by approximately 57.6% and 62.5% in the liver at 2 h and 6 h, respectively, and by approximately 70.8% in the kidneys at 2 h. This “high placenta, low fetus” distribution is particularly critical for use in pregnancy, as it suggests that the risk of potential adverse fetal exposure may be reduced while increasing local placental exposure.

To further quantify targeting, this study calculated the tissue-to-plasma partition coefficient (Kp; tissue mean/plasma concentration at the same time point) at 2 h. As shown in Table 2, placental Kp in the A-RGDV group increased markedly from 0.015 to 0.786, approximately a 50-fold increase. In addition, the fetus-to-placenta ratio decreased substantially from 0.468 to 0.002. These relative distribution metrics strengthened the conclusion that A-RGDV may achieve enrichment through placenta-specific binding or local release, while reducing off-target exposure overall.

**Table 2:** Targeted quantitative indicators at 2 h after different treatment.

Drug	Kp (Placenta/Plasma)	Fetus/Placenta
Aspirin	0.015	0.468
A-RGDV	0.786	0.002

A central translational challenge in pregnancy therapeutics is achieving meaningful drug exposure at the maternal-fetal interface while minimizing fetal exposure. Low-dose aspirin is widely recommended for preeclampsia prevention, yet its benefit is largely prophylactic and may be limited by insufficient placental delivery once disease is established, while systemic exposure raises tolerability and safety considerations during prolonged administration (Committee, 2018). In this context, the most notable advantage of A-RGDV is not an exaggerated early peak, but a reshaped exposure profile together with a pronounced redistribution toward the placenta. Quantitative LC-MS/MS analysis showed robust placental enrichment while fetal levels were reduced, indicating that A-RGDV does not simply increase global exposure but preferentially concentrates aspirin-related exposure at the maternal-fetal interface. We speculate that this pattern reflects a combination of integrin-guided retention at integrin-rich placental compartments and formulation-dependent release/hydrolysis kinetics that prolong the effective exposure window. Such placenta-selective delivery is particularly meaningful for pregnancy therapeutics, as it can decouple therapeutic benefit from systemic burden and potentially mitigate fetal exposure.

Notably, salicylic acid was used as a surrogate due to the rapid hydrolysis of aspirin, which is a common analytical approach in aspirin PK studies (Angiolillo et al., 2019; Vinogradov et al., 2025). However, simultaneous quantification of the parent compound and metabolite in maternal and placental microenvironments will further refine mechanistic interpretation in future work.

### 3.10 Therapeutic effects of A-RGDV in the pre-eclampsia mice

**Fig. 6:**
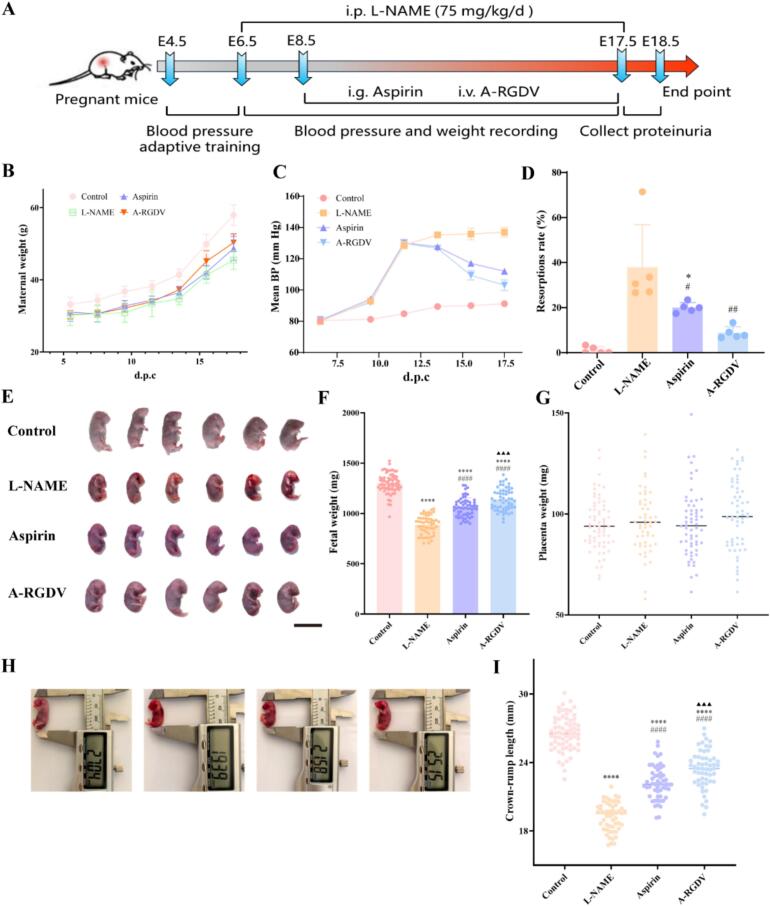
(A) Schematic of the L-NAME-induced PE mouse model and treatment schedule (L-NAME 6.5–17.5 d.p.c., aspirin or A-RGDV 8.5–17.5 d.p.c.). (B) Body weight of ICR pregnant mice in different treatment groups (n = 5). (C) Mean BP of ICR pregnant mice in different treatment groups. (D) The resorptions rate of ICR pregnant mice in different treatment groups (n = 5). (E) The surviving embryo photograph of pregnant mice in different groups. Scale bars, 20 mm. (F) Fetal weight of pregnant mice in different treatment groups (n = 5). (G) The placenta weight of pregnant mice in different treatment groups (n = 5). (H) The image of caliper measurement of fetuses from each group. (I) Fetal crown-rump length (n = 5). * *p* < 0.05, **** *p* < 0.0001 *vs* Control group, # *p* < 0.05, ## *p* < 0.01, #### *p* < 0.0001 *vs* L-NAME group, ▲▲▲ *p* < 0.001 *vs* Aspirin-treatment group.

In light of the favorable pharmacokinetic behavior and placental tissue distribution, this study next assessed the therapeutic efficacy of A-RGDV in a murine model of PE. The study design and treatment allocation are shown in Fig. 6A. In preclinical PE research, L-NAME-induced hypertension is a widely used model, pilot experiments established that intraperitoneal L-NAME at 75 mg·kg^−1^·day^−1^ induces a PE-like phenotype in 80% of pregnant ICR mice while maintaining maternal survival. To ensure robust model establishment prior to therapy, the pregnant mice received L-NAME for 2 consecutive days before drug intervention. For pharmacodynamic evaluation, treatment was administered from 8.5 to 16.5 d.p.c., a window selected based on repeated pilot trials to capture the period of active placentation and spiral-artery remodeling while terminating before impending parturition to maximize fetal yield for endpoint analyses.

In the L-NAME-induced PE model, maternal blood pressure, fetal development, and placental growth were markedly impaired compared with normal pregnancy. Mean blood pressure (MBP) increased progressively during gestation in the model group, confirming successful PE induction. As shown in Fig. 6B, body-weight gain during gestation was also blunted in the model, consistent with PE-associated fetal growth restriction. Both aspirin and A-RGDV partially restored maternal weight trajectories, with A-RGDV most closely approximating the normal curve. Antihypertensive responses were observed in both treatment groups, however, A-RGDV produced a more pronounced and sustained MBP reduction, approaching control levels within several days of dosing (Fig. 6C), suggesting improved bioavailability and placental targeting relative to free aspirin.

Fetal outcomes mirrored these benefits. The resorption rates in the model, aspirin, and A-RGDV groups were 37.83%, 20.00%, and 8.79%, respectively, with A-RGDV showing the best efficacy (Fig. 6D). At the end of the experiment, our group recorded the weights of viable fetuses and placentas for each pregnant mouse. Fig. 6E-F illustrated the live fetuses in the different groups. Compared with normal pregnancy, fetal weight in untreated PE pregnant mice was significantly reduced, consistent with fetal growth restriction, whereas A-RGDV increased fetal weight more markedly than aspirin (*p* < 0.001). Placental weights did not differ greatly among groups, subsequent assessment by histology and placental factors was performed to evaluate qualitative improvements (Fig. 6G). The embryo-to-placenta weight ratio (FPR) is considered an important indicator of pregnancy (Hayward et al., 2016). In PE, the FPR tends to be reduced, suggesting that decreased placental efficiency and maladaptation of placental development or perfusion are strongly associated with an increased risk of adverse pregnancy outcomes (Phaloprakarn et al., 2025). In our study, both aspirin- and A-RGDV-treated pregnant mice had improved FPR compared to the L-NAME group (Fig. S5). Notably, A-RGDV produced a more pronounced improvement and approached the normal control, indicating a more effective restoration of placental function than aspirin (*p* < 0.01). This pattern aligned with lower maternal blood pressure, reduced resorption rate, and improved fetal weight and size, underscoring the broader benefits of A-RGDV on pregnancy outcomes. Representative fetal image showed severe growth restriction and resorption in the L-NAME group, which were substantially rescued by A-RGDV but only partially improved by aspirin, indicating that A-RGDV enhances fetal viability and morphological development (Fig. S6). Consistent with these findings, crown-rump length measurements of fetuses further corroborated this conclusion (Fig. 6H-I).

### 3.11 *In vivo* angiogenesis evaluation

**Fig. 7:**
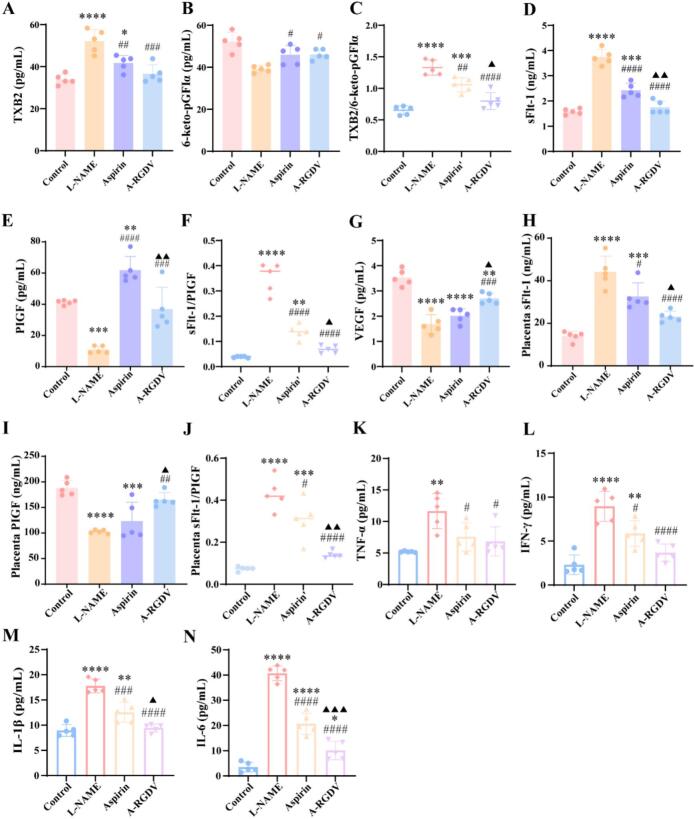
The concentration of (A) TXB_2_, (B) 6-keto-pGFIα, and (C) TXB_2_/ 6-keto-pGFIα in serum in different treatment groups (n = 5). The concentration of (D) sFlt-1, (E) PIGF, and (F) sFlt-1/PIGF in serum in different treatment groups (n = 5). (G) The concentration of VEGF in serum in different treatment groups (n = 5). (G) The concentration of endotoxin in amniotic fluid in different treatment groups (n = 5). The concentration of (H) sFlt-1, (I) PIGF, and (J) sFlt-1/PIGF in placenta in different treatment groups (n = 5). The concentration of (K) TNF-α，(L) IFN-γ, (M) IL-1β, and (N) IL-6 in serum in different treatment groups (n = 5). * *p* < 0.05, ** *p* < 0.01, *** *p* < 0.001, **** *p* < 0.0001 *vs* Control group, # *p* < 0.05, ## *p* < 0.01, ### *p* < 0.001, #### *p* < 0.0001 *vs* L-NAME group, ▲ *p* < 0.05, ▲▲ *p* < 0.01, ▲▲▲ *p* < 0.001 *vs* Aspirin-treatment group.

Maternal serum and placental analytes were quantified to assess vascular and angiogenic status in normal pregnancy, the L-NAME induced PE model, and treatment groups. We profiled vasoactive mediators linked to endothelial tone. The model group showed elevated TXB₂, the stable metabolite of the vasoconstrictor thromboxane A₂, together with reduced prostacyclin-associated signal, indicating a shift toward vasoconstriction and platelet activation (Fig. 7A-B). Consistent with these individual mediators, the composite serum TXB₂/6-keto-PGF₁α ratio was 1.330 ± 0.121 in the model and declined to 1.057 ± 0.114 with aspirin, whereas A-RGDV lowered it further to 0.798 ± 0.134 (Fig. 7C), indicating a stronger restoration of prostanoid balance by A-RGDV. Aspirin partially corrected this thromboxane/prostacyclin imbalance, consistent with its COX-dependent antiplatelet activity; importantly, A-RGDV produced a similar or greater improvement, decreasing TXB₂ and enhancing prostacyclin-associated tone toward normal. In the L-NAME model, we observed a classic PE-like profile characterized by suppression of the proangiogenic factors placental growth factor (PlGF) and vascular endothelial growth factor (VEGF), together with an increase in the antiangiogenic decoy receptor sFlt-1 in maternal serum. This antiangiogenic imbalance is known to impair endothelial function, promote vasoconstriction, and drive maternal hypertension (Zhang et al., 2025). Treatment with A-RGDV partially corrected the antiangiogenic shift, increasing maternal PlGF and VEGF while reducing sFlt-1 levels relative to the untreated L-NAME group (Fig. 7D-G). In line with these single-analyte trends, the serum sFlt-1/PlGF ratio fell from 0.352 ± 0.060 in the model to 0.138 ± 0.028 with aspirin, and to 0.068 ± 0.015 with A-RGDV (Fig. 7F), again showing a greater normalization with A-RGDV than with aspirin. A similar trend was observed in placental tissue, where A-RGDV restored PlGF and lowered placental sFlt-1 more effectively than aspirin (Fig. 7H-J).

Functionally, these biochemical changes translated into improved *in vivo* outcomes. Relative to the untreated model, A-RGDV reduced maternal blood pressure and improved placental and fetal growth indices, including placental and fetal weights, crown-rump length, and fetal resorption, with favorable trends compared with aspirin in several measures. Taken together, these data indicated that A-RGDV not only alleviated maternal hypertension but also rebalanced the local maternal-fetal environment by i) restoring a proangiogenic PlGF/VEGF *vs* sFlt-1 profile and ii) normalizing vasoactive signaling. We propose that this benefit derives from placenta-targeted delivery *via* the RGDV motif, which supports trophoblast survival, migration, and spiral artery remodeling, in combination with the anti-inflammatory and antiplatelet actions of the aspirin payload. Thus, A-RGDV acts at both the placental and systemic levels, offering broader therapeutic correction of PE-associated pathophysiology than aspirin alone.

Recent therapeutic strategies have begun to directly modulate placental angiogenesis using advanced delivery systems. For example, placenta-tropic VEGF mRNA lipid nanoparticles have been reported to ameliorate murine preeclampsia and partially restore placental vasculature while improving systemic biomarkers (Swingle et al., 2025). Compared with such gene-based approaches that directly supplement a pro-angiogenic cue, A-RGDV represents a small-molecule based, ligand-guided strategy that leverages placenta-selective delivery to enhance local bioactivity and indirectly restore angiogenic homeostasis by alleviating placental stress, inflammation, and vasoactive imbalance. This distinction may be clinically meaningful, because placenta-resident delivery systems are increasingly recognized as a rational way to minimize fetal exposure while reducing maternal off-target effects in placenta-originated diseases (Tang et al., 2023). Taken together, our results support a mechanistic sequence whereby placenta-favored delivery enhances aspirin-related activity locally, mitigates placental inflammatory stress, and enables partial restoration of angiogenic balance and vascular remodeling. This provides a coherent explanation for the superior efficacy of A-RGDV over free aspirin at matched dose equivalents.

### 3.12 Inflammatory response evaluation

This study also measured circulating proinflammatory cytokines in maternal serum from normal pregnant mice, L-NAME induced PE model mice, and mice treated with aspirin or A-RGDV. In all four panels, the PE model group showed a marked increase in inflammatory mediators compared with the normal pregnancy group, indicating that L-NAME induction triggered a systemic inflammatory state characterized by elevated Th1/proinflammatory signaling. This is consistent with the known pathophysiology of PE, in which placental ischemia and endothelial dysfunction drive maternal inflammatory activation. Both aspirin and A-RGDV reduced cytokine levels relative to the untreated PE model. A-RGDV lowered key cytokines (including TNF-α and IL-6) toward or near baseline more effectively than aspirin, and also limited IL-1β and IFN-γ production, suggesting a broader suppression of the proinflammatory milieu (Fig. 7K-N). Taken together, these data indicated that A-RGDV not only improved hemodynamic and placental endpoints, but also attenuated the maternal systemic inflammatory response associated with PE. Because excessive TNF-α and IL-6 signaling contributed to endothelial injury, vasoconstriction, and impaired uteroplacental perfusion, the anti-inflammatory effect of A-RGDV likely contributed directly to its antihypertensive action and to the observed improvements in fetal growth and survival.

### 3.13 Histological and vascular assessment of the placenta *in vivo*

**Fig. 8:**
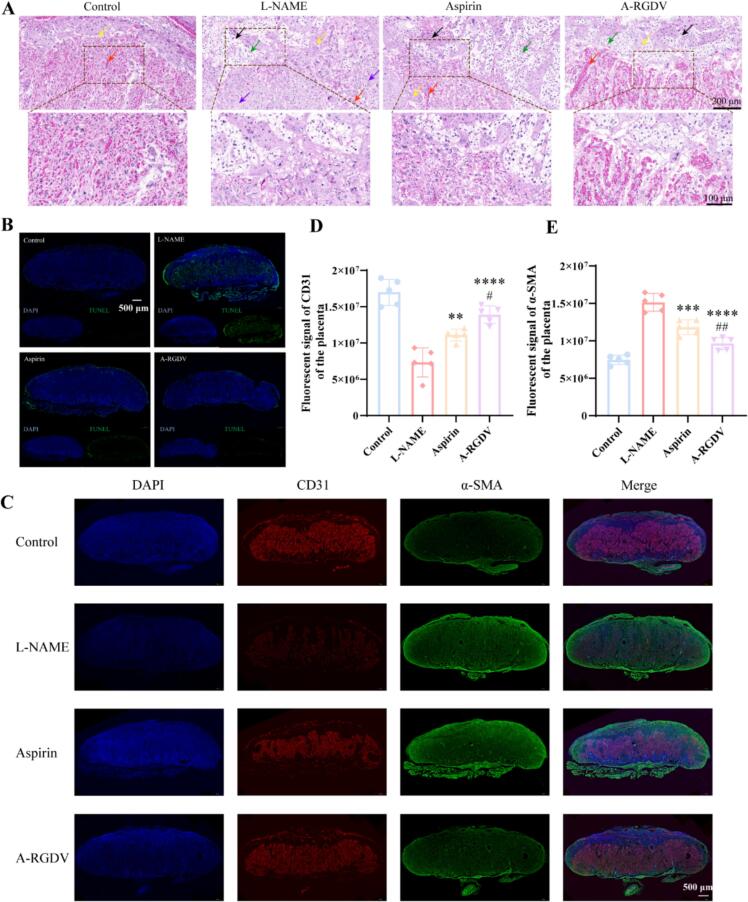
(A) Representative H&E-stained sections of placentas from Control, L-NAME, L-NAME + aspirin, and L-NAME + A-RGDV groups. Red arrow denoted blood sinuses; Yellow arrow denoted nourishing cells; Green arrow denoted glycogen cells; Black arrow denoted cellular necrosis; Purple arrow denoted tissue necrosis. (B) Whole-placenta TUNEL staining (green) with DAPI (blue) showing extensive apoptotic nuclei in L-NAME placentas, reduced apoptosis with aspirin, and markedly attenuated apoptosis with A-RGDV. (C) Representative images of CD31 (endothelial marker, red) and α-SMA (smooth muscle marker, green) double immunofluorescence with DAPI (blue). (D) Integrated fluorescent signal for CD31 of the placenta (n = 5). (E) Integrated fluorescent signal for α-SMA of the placenta (n = 5). ** *p* < 0.01, *** *p* < 0.001, **** *p* < 0.0001 *vs* Control group, # *p* < 0.05, ## *p* < 0.01 *vs* L-NAME group. (For interpretation of the references to colour in this figure legend, the reader is referred to the web version of this article.)

As shown in Fig. 8A, an intact placental architecture in the control group, with patent maternal blood sinuses (red arrows), abundant nourishing trophoblasts (yellow arrows), and glycogen cells (green arrows), and no obvious necrosis. L-NAME treatment group markedly disrupted the placenta, such as blood sinuses became narrowed or collapsed, nourishing cells were damaged, glycogen cells were reduced, and both cellular necrosis (black arrows) and tissue necrosis (purple arrows) were prominent, consistent with poor perfusion and tissue injury. The aspirin treatment group partially rescued these changes, reopening some blood sinuses and reducing necrotic areas, although the structure remained irregular. The A-RGDV treatment group provided the greatest improvement, with widely open sinuses, better preservation of nourishing and glycogen cells, and minimal necrosis, making the overall architecture close to that of controls, indicating restoration of microcirculation and tissue integrity. Consistent with these structural findings, TUNEL staining revealed minimal apoptosis in controls, a diffuse and intense apoptotic signal across the labyrinthine zone in L-NAME placentas, a clear reduction after aspirin, and a further reduction after A-RGDV to near-baseline levels, indicating effective suppression of trophoblast cell death (Fig. 8B).

Vascular phenotypes assessed by CD31^+^/α-SMA double immunofluorescence showed a sparse (Fig. 8C), fragmented CD31^+^ endothelial network and aberrant extension of α-SMA into the labyrinth after L-NAME, consistent with impaired micro-angiogenesis and pathological arteriolar muscularization. Aspirin partially restored endothelial continuity and curtailed α-SMA spread. A-RGDV yielded the most normalized pattern, characterized by a dense and uniform CD31^+^ capillary network and α-SMA largely confined to larger feeding vessels and septa, indicative of preserved capillary architecture and more physiological vascular maturation. Analysis of the fluorescence intensity also testified that A-RGDV improved the state of the placental vasculature in PE (Fig. 8D-E).

Together, the concordant improvements in tissue morphology, reduced apoptotic burden, and normalization of vascular remodeling demonstrate that A-RGDV confers robust placental protection *in vivo* and outperforms low-dose aspirin under the same experimental conditions. These data support A-RGDV as a promising therapeutic candidate for placental repair in PE.

### 3.14 Biosafety evaluation

**Fig. 9:**
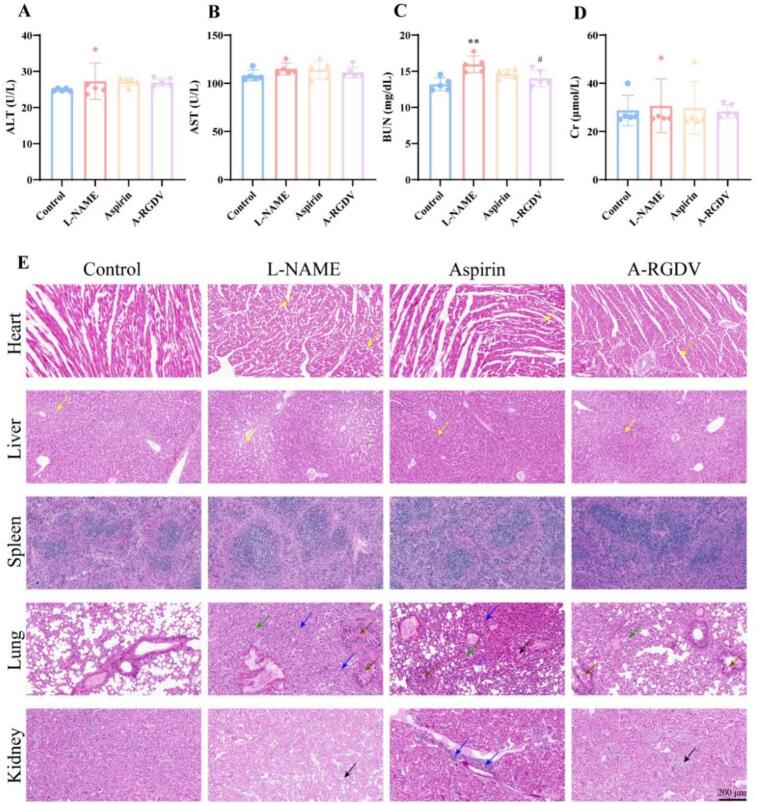
The concentration of (A) ALT, (B) AST, (C) BUN, and (D) Cr in serum in different treatment groups (n = 5). (E) Representative H&E staining of heart, liver, spleen, lung, and kidney tissues. In the heart, the yellow arrow denoted vacuolar degeneration of cardiomyocytes. In the liver, the yellow arrow denoted hepatic steatosis. In the lung, the green arrow denoted alveolar narrowing, the blue arrow denoted granulocyte infiltration, the brown arrow denoted epithelial cell sloughing, and the black arrow denoted eosinophilic material. In the kidney, the black arrow denoted epithelial cell necrosis, the blue arrow denoted inflammatory cell infiltration. ** *p* < 0.01 *vs* Control group, # *p* < 0.05 *vs* L-NAME group. (For interpretation of the references to colour in this figure legend, the reader is referred to the web version of this article.)

At the experimental endpoint, maternal liver and kidney function were assessed by measuring serum ALT, AST, BUN, and Cr across all treatment groups. For hepatic indexes **(**Fig. 9A-B), the PE model group showed no marked elevation compared with normal pregnancy, indicating that L-NAME at the dose used did not produce overt hepatocellular injury. ALT and AST levels in the A-RGDV-treated mice remained comparable to both the normal and aspirin groups. Although there was mild variability between individual animals, there was no systematic upward shift after A-RGDV administration. This suggests that neither free aspirin nor A-RGDV caused detectable hepatotoxicity under the dosing schedule used.

A similar pattern was observed for renal function (Fig. 9C-D). The L-NAME model group showed a slight increase in BUN, which may be associated with proteinuria in PE, however, BUN and Cr levels remained within a range that did not indicate overt renal failure at the examined gestational time point. Treatment with A-RGDV did not worsen either parameter: BUN and Cr in the A-RGDV group were essentially within the same range as the normal pregnant and aspirin-treated groups. There was no signal of uremia or impaired glomerular filtration associated with A-RGDV.

Histological examination showed intact structures of the heart, liver, spleen, lung, and kidney in the normal group (Fig. 9E). Compared with the normal group, the L-NAME group exhibited clear pathological changes in some tissues, including marked granulocyte infiltration and moderate thickening of the alveolar septa in the lung, as well as mild renal tubular injury in the kidney. Aspirin treatment partially alleviated these lesions, but some abnormalities were still observed. Overall, the A-RGDV-treated group showed milder pathological changes, with markedly reduced lung inflammation and relatively preserved kidney architecture.

Taken together with the serum biochemical findings, these results indicate that A-RGDV exhibits acceptable maternal hepatorenal safety under the tested conditions and may mitigate L-NAME-induced tissue injury. In the PE model, A-RGDV improved blood pressure control, placental angiogenic balance, and fetal outcomes without increasing hepatic or renal burden, indicating that its therapeutic benefits are not achieved at the expense of maternal organ toxicity.

## 4 Conclusion

In summary, this study reported a placenta-targeted, nanosized aspirin-RGDV conjugate designed to overcome key clinical limitations of aspirin in established PE therapy. Through ligand-guided targeting and nanoscale formulation, A-RGDV achieved sustained and preferential placental exposure while minimizing fetal and off-target organ distribution. This optimized biodistribution translated into superior therapeutic outcomes, including improved placental perfusion, restoration of microvascular architecture, normalization of angiogenic and vasoactive signaling, and attenuation of inflammatory and oxidative stress responses, all without increasing systemic peak exposure or maternal hepatorenal burden. Importantly, by decoupling therapeutic benefit from prolonged systemic exposure, A-RGDV overcame major constraints of long-term low-dose aspirin therapy in PE. Collectively, these findings demonstrate A-RGDV as a rational and translational placenta-oriented therapeutic strategy, advancing a safer and more effective paradigm for the management of PE.

## Funding

This study was financially supported by the National Health Commission Scientific Research Fund−Major Science and Technology Plan Project of Zhejiang Province (Project number: WKJ-ZJ-2533 and WKJ-ZJ-26050), Zhejiang Provincial Natural Science Foundation of China (Grant No. LQN25H270002), and Chronic Disease Management Research Project of National Health Commission Capacity Building and Continuing Education Center (Grant No. GWJJMB202510041003).

## Ethics approval

All animal experiment protocols were approved by the Scientific Investigation Board of Zhejiang Chinese Medical University (approval number IACUC-20251110-05).

## CRediT authorship contribution statement

**Ying Zhang:** Writing – original draft, Methodology, Investigation, Data curation. **Wenqiang Qian:** Writing – review & editing, Supervision, Software, Investigation, Data curation. **Yao Yao:** Writing – review & editing, Investigation, Data curation. **Dongli Sun:** Writing – review & editing, Investigation, Funding acquisition, Formal analysis, Data curation. **Zhiyuan Ma:** Writing – review & editing, Investigation, Funding acquisition, Formal analysis. **Xian Zhang:** Writing – review & editing, Funding acquisition, Formal analysis. **Huidi Jiang:** Writing – review & editing, Investigation, Data curation. **Tian Dong:** Writing – review & editing, Data curation. **Weidong Fei:** Writing – review & editing, Supervision, Investigation, Funding acquisition, Formal analysis, Data curation. **Caihong Zheng:** Supervision, Project administration, Methodology, Formal analysis.

## Declaration of competing interest

The authors declare that they have no known competing financial interests or personal relationships that could have appeared to influence the work reported in this paper.

## Data Availability

Data will be made available on request.

## Appendix A Supplementary data

Supplementary material Image 1
