# Supplementary material for: Placenta-oriented self-assembled Aspirin-RGDV nanoconjugates attenuate preeclampsia through restoration of angiogenic balance
Source: Int J Pharm X. 2026 Apr 16;11:100545. doi: 10.1016/j.ijpx.2026.100545 (PMC13101701; doi:10.1016/j.ijpx.2026.100545)
Supplement: Supplementary file 1 — Supplementary material [file mmc1.docx]

**Supplemental information**

**Placenta-oriented Self-assembled Aspirin-RGDV Nanoconjugates Attenuate Preeclampsia through Restoration of Angiogenic Balance**

Ying Zhang ^1, #^, Wenqiang Qian ^2, #^, Yao Yao ^2^, Dongli Sun ^2^, Zhiyuan Ma ^2^, Xian Zhang ^2^, Huidi Jiang ^1^, Tian Dong ^2^, Weidong Fei ^2,^ *, Caihong Zheng ^2,^ *

^1^ Research Center for Clinical Pharmacy, College of Pharmaceutical Sciences, Zhejiang University, Hangzhou 310058, China

^2^ Women’s Hospital, Zhejiang University School of Medicine, Hangzhou 310006, China

^#^ These authors contributed equally to this work.

*Corresponding authors: feiweidong@zju.edu.cn (W. Fei) and [chzheng@zju.edu.cn](mailto:chzheng@zju.edu.cn) (C. Zheng).


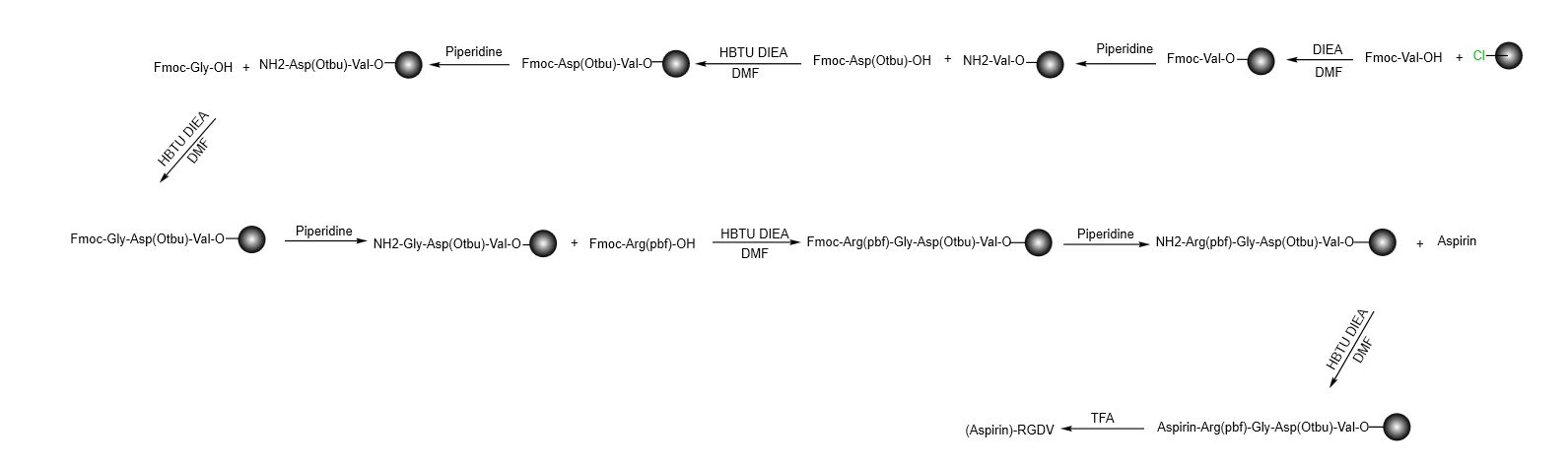


**Fig. S1.** Synthetic route of A-RGDV.


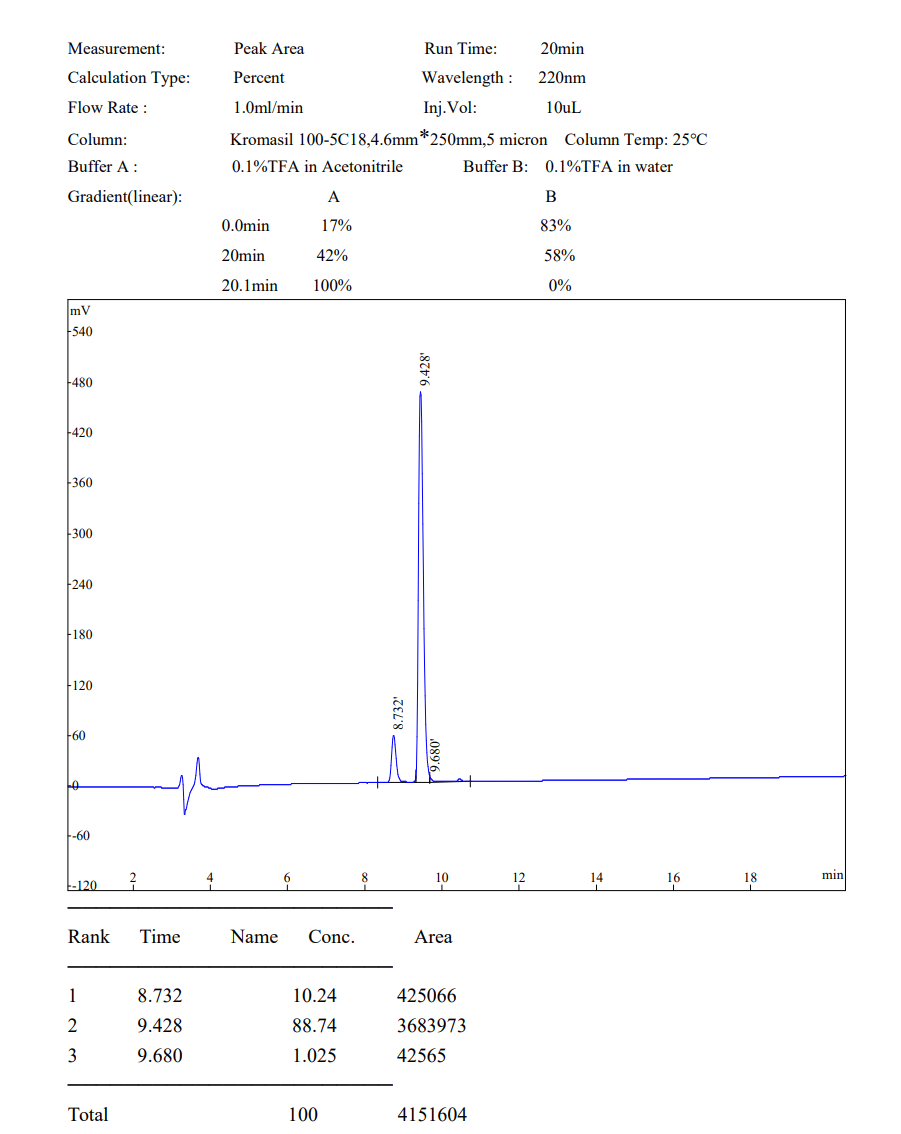


**Fig. S2.** HPLC analysis report of A-RGDV.


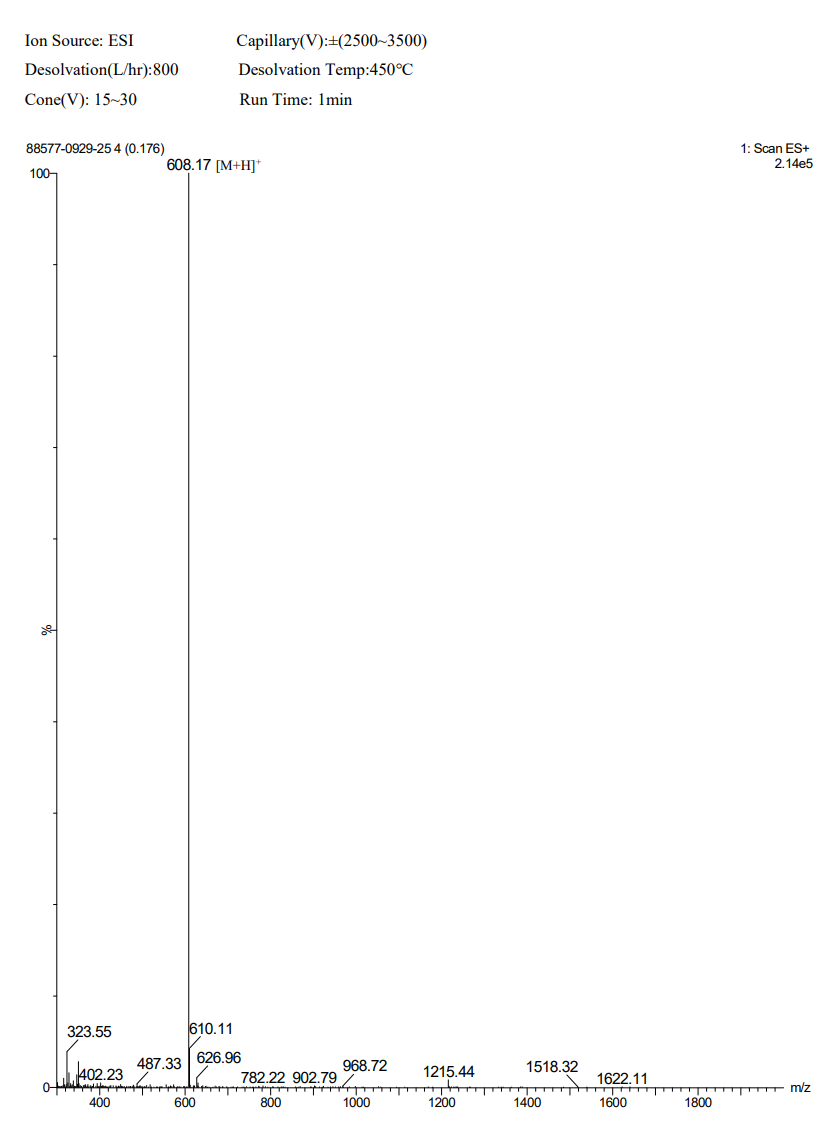


**Fig. S3.** MS analysis report of A-RGDV.


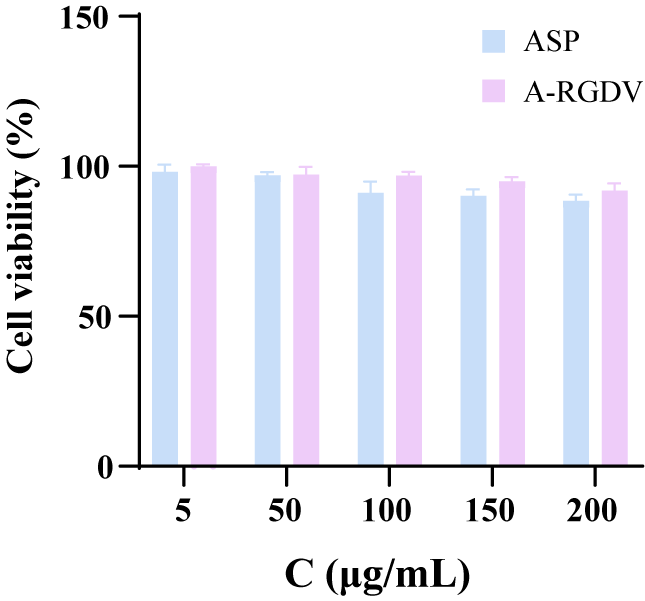


**Fig. S4.** Cytotoxicity of aspirin solution, or A-RGDV on HTR-8/SVneo cells after incubation for 24 h (n = 5).


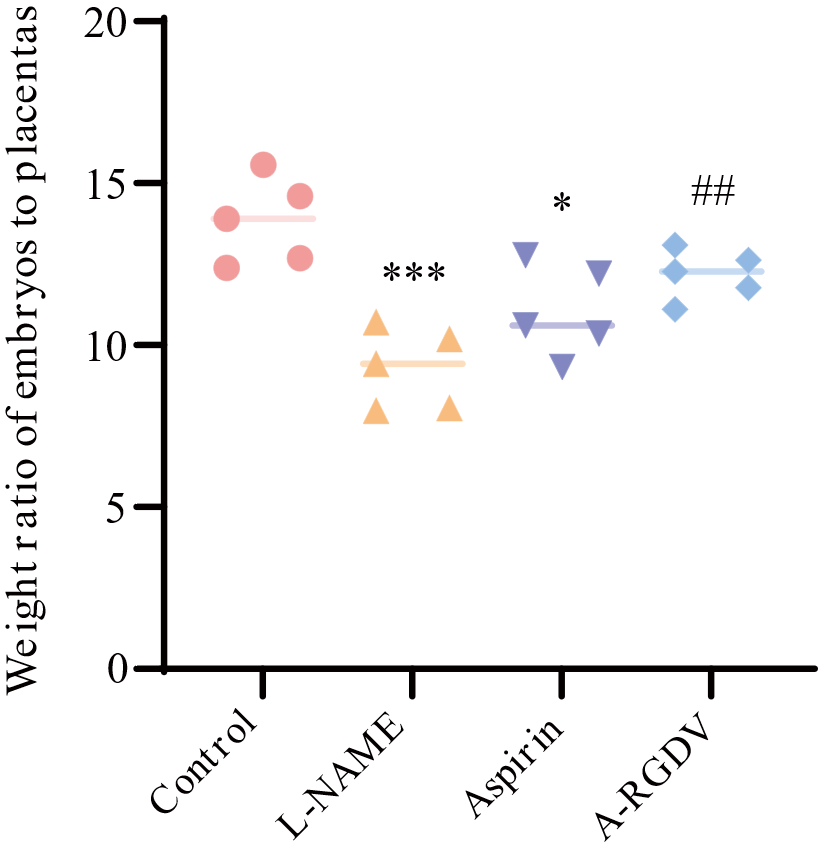


**Fig. S5.** Weight ratio of embryos to placentas in each group (n = 5). * *p* < 0.05, *** *p* < 0.001 *vs* Control group, ## *p* < 0.01 *vs* L-NAME group.


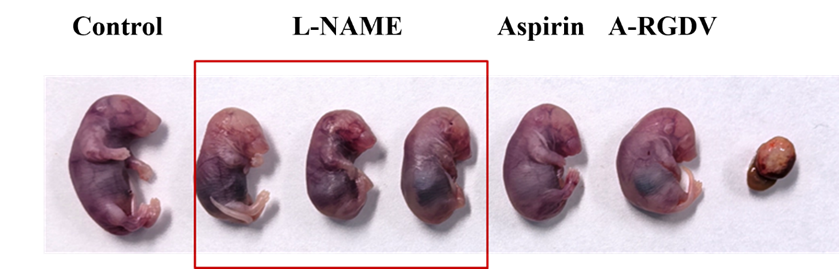


**Fig. S6.** Representative image of fetuses from each group.
